# Toughening Immiscible Polymer Blends: The Role of Interface-Crystallization-Induced Compatibilization Explored Through Nanoscale Visualization

**DOI:** 10.1021/acsami.4c10829

**Published:** 2024-10-16

**Authors:** Hamid Ahmadi, Paul M. H. van Heugten, Alexander Veber, Ljiljana Puskar, Patrick D. Anderson, Ruth Cardinaels

**Affiliations:** † Processing and Performance of Materials, Department of Mechanical Engineering, Eindhoven University of Technology, P.O. Box 513, Eindhoven 5600 MB, The Netherlands; ‡ Department of Chemistry, Humboldt-Universität zu Berlin, Brook-Taylor-Straße 2, Berlin 12489, Germany; § Institute for Electronic Structure Dynamics, Helmholtz-Zentrum Berlin für Materialien und Energie GmbH, Albert-Einstein-Straße 15, Berlin 12489, Germany; ∥ Soft Matter, Rheology and Technology, Department of Chemical Engineering, KU Leuven, Celestijnenlaan 200J, Leuven 3001, Belgium

**Keywords:** immiscible polymer blend, compatibilization, nano-IR, stereocomplex crystallization, interlayer thickness, interfacial crystallization

## Abstract

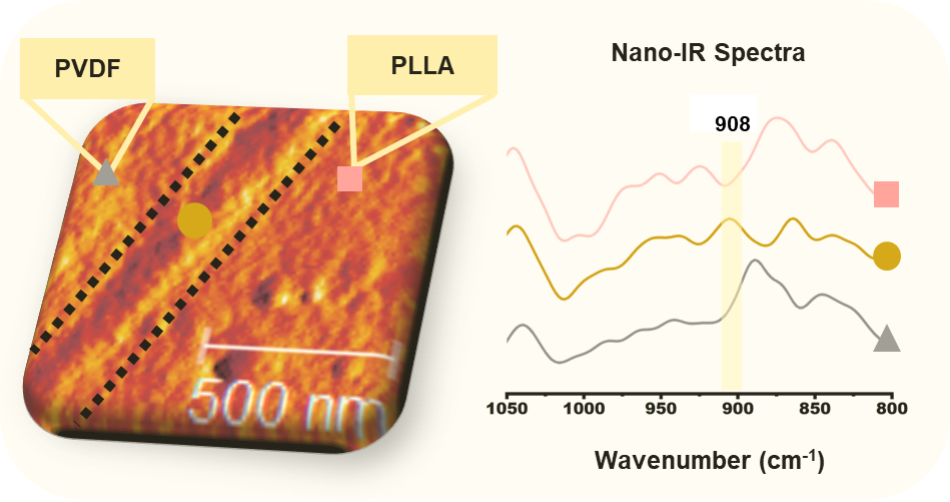

This study explores the novel approach of interface-crystallization-induced compatibilization (ICIC) via stereocomplexation as a promising method to improve the interfacial strength in thermodynamically immiscible polymers. Herein, two distinct reactive interfacial compatibilizers, poly(styrene-*co*-glycidyl methacrylate)-*graft*-poly(l-lactic acid) (SAL) and poly(styrene-*co*-glycidyl methacrylate)-*graft*-poly(d-lactic acid) (SAD) are synthesized via reactive melt blending in an integrated grafting and blending process. This approach is demonstrated to enhance the interfacial strength of immiscible polyvinylidene fluoride/poly l-lactic acid (PVDF/PLLA) 50/50 blends via ICIC. IR nanoimaging indicates a cocontinuous morphology in the blends. The blend compatibilized with SAD exhibits a higher storage modulus, as unveiled by small amplitude oscillatory shear (SAOS) in the melt state at a temperature below the melting temperature of the stereocomplex (SC) crystals and by DMTA measurements in the solid state. This increase is attributed to the formation of a 200–300 nm thick rigid interfacial SC crystalline layer that is directly visible using AFM imaging and chemically characterized via IR nanospectroscopy. This ICIC also results in a significant toughening of the blend, with the elongation at break increasing more than 20-fold. Moreover, the fracture toughness factor obtained from single edge-notch bending (SENB) tests is doubled with ICIC as compared to the uncompatibilized blend, indicating the strong crack-resistance capability as a result of ICIC. This improvement is also evident in SEM images, where thinner and longer fibrillation is observed on the fractured surface in the presence of ICIC.

## Introduction

Blending immiscible polymers is a versatile technique for producing hybrid materials that exhibit a balanced interplay of favorable properties inherited from each component. The existence of weak interfacial adhesion and unstable morphologies, arising from a robust thermodynamic driving force for polymer demixing, presents challenges in both the processing and final performance of these materials.^1^ To accomplish the maximum potential of immiscible polymer blends, it is imperative to tailor both the polymer–polymer interface and the blend morphology.^2^ Many approaches have been established to improve the morphology stabilization and interfacial adhesion in immiscible polymer blends, such as using premade or reactive compatibilizers,^3^ introducing a thin intermediate layer at the blend interface,^4,5^ and localization of nanoparticles at the interface,^1,6,7^ In addition, the novel approach of interface-crystallization-induced compatibilization (ICIC) has recently garnered attention as a promising method to enhance the interfacial strength between thermodynamically immiscible polymers.^9−11^ ICIC is usually achieved through the formation of stereocomplex (SC) crystals at the interface between polymer chains with inverse chirality such as polylactic acid (PLA) enantiomers, i.e., poly l-lactic acid (PLLA) and poly d-lactic acid (PDLA).^9−21^

Various practical strategies have been employed to attain ICIC through stereocomplexation in immiscible blends. In PLLA-based blends, PDLA is reactively grafted onto chains that are the same or compatible with the second blend component.^9−18,21^ In other immiscible blends, both PDLA and PLLA can be reactively grafted onto the main chains of the blend components.^19,20^ The second copolymer segment can form physical entanglements or undergo chemical reactions with the respective phase of the blend. Interfacially positioned nanorods containing PDLA grafts have also been utilized to form SC crystals at the interface of a PLLA-based blend.^21^ In all of these strategies, SC crystals are formed due to hydrogen-bonding forces between PLLA and PDLA.^22^ SC crystals exhibit a dense chain packing which results in a higher melting point and higher modulus as compared to PLA homocrystals.^23,24^ Accordingly, the formed SC crystals at the interface both strengthen the interface of the polymer blends and enhance other thermomechanical properties.

The cocontinuity and interfacial interactions in cocontinuous blends are crucial for enhancing mechanical properties by mitigating stress concentrations, especially in cases involving modulus mismatch between the blend components.^4^ ICIC effectively redistributes stresses at the interface and thereby consequently prevents interfacial failure and crack propagation.^12^ For 3D-printed filaments, it has been demonstrated that SC crystals formed at the interface between the filaments enhance interfacial stiffness and improve thermo-mechanical performance.^25^ Similarly, it has also been shown that the rigid SC crystalline layer strengthens the interface of polymer blends, which leads to an improvement in the mechanical properties of the blend.^12,14,15^ The formation of SC crystals solely at the interface is crucial to achieve a high physical performance of the blend using ICIC.^21^ As previously noted, to attain interfacial compatibilization via stereocomplexation, PLLA and PDLA are initially grafted through reactive blending onto a component that is either identical to or compatible with the representative phase of the immiscible blend. Components formed during reactive blending can readily migrate away from the interface due to their asymmetric structure or the affinity of a portion of the copolymer for both phases of the blend.^26^ This migration may result in the crystalline phase forming in a location other than the interface, thereby failing to impart the desired final properties. Therefore, it is crucial to adopt an appropriate approach to achieve ICIC.

The concept of ICIC through stereocomplexation is well established in the literature; experimentally identifying the locations and distributions of the formed SC crystals in a blend is challenging. In the majority of the available literature, the crystalline layer in ICIC has been explored by using methods such as atomic force microscopy (AFM), mechanical, and rheological techniques. However, these methods do not provide direct evidence of interfacial SC formation as a consequence of the ICIC approach. Chen et al.^11^ and Wang et al.^10^ employed nanomechanical mapping based on AFM to analyze the location and distribution of SC crystals in PBAT/PLLA and PVDF/PLLA blends, respectively, with a PDLA-containing copolymer. However, AFM can not provide chemical information about the materials, which is essential for unambiguous determination of the interfacial location of ICIC. In addition, it is more challenging to use AFM for the characterization of ICIC in semicrystalline blends, wherein the mechanical properties of the crystalline phase of the components are close to that of the SC crystals. Fourier-transform infrared (FTIR) spectroscopy is capable of gaining information about the chemical composition of components. Unfortunately, the spatial resolution of the commonly used far-field infrared spectroscopy methods is limited to approximately 5 μm due to the diffraction limit. The crystalline size of ICIC is on the nanometer scale;^10,11^ therefore, its direct chemical characterization requires the use of advanced analytical techniques capable of performing measurements at nanometer spatial scales. Near-field infrared spectroscopy techniques, such as AFM-IR and infrared scattering optical microscopy (s-SNOM), have been previously utilized in the study of polymer blends.^28−34^ Nano-IR techniques enable both imaging and chemical characterization of complex materials with spatial resolution far beyond the diffraction limit of infrared light. The spatial resolution is on the order of the AFM tip radius, and a resolution down to 10 nm can be achieved.^35−37^ These nano-IR techniques allow chemical characterization of the sample surface at a nanoscale resolution, which is crucial for ICIC characterization. Nevertheless, the application of neither AFM-IR nor IR s-SNOM techniques to characterize ICIC and the interfacial crystalline layer in immiscible blends has not been demonstrated up to now.

In this study, two distinct reactive interfacial compatibilizers, poly(styrene-*co*-glycidyl methacrylate)-*graft*-poly(l-lactic acid) (SAL) and poly(styrene-*co*-glycidyl methacrylate)-*graft*-poly(d-lactic acid) (SAD), are synthesized via reactive melt blending and are subsequently employed to achieve ICIC in immiscible PVDF/(PLLA) 50/50 blends. The SAN-based copolymer is specifically selected in this study for its exclusive miscibility in PVDF, whereas previously used poly(methyl methacrylate)-based copolymers have affinity toward both polymers.^10^ The integrated process, including both grafting and blending, which is utilized in the present work, is particularly advantageous for commercial production. s-SNOM coupled to a broadband synchrotron infrared light source is used to characterize the blend morphology and visualize SC formation at the blend interface as well as to map out its chemical fingerprint. The effects of ICIC via stereocomplexation on the rheological properties, crystallization behavior, and mechanical properties are also investigated. This study offers new perspectives and practical methods for understanding how the strength of interfaces in thermodynamically immiscible polymer blends can be improved through ICIC.

## Materials and Methods

### Materials

Poly(vinylidene fluoride) (Kynar 720, *M*_w_ = 210 kg/mol, PDI = 2, further termed PVDF) was supplied by Arkema (France). Two commercially available polylactic acids, namely, poly(l-lactic acid) (Luminy-L175, *M*_w_ = 208 kg/mol, PDI = 2.41, further referred to as PLLA) and poly(d-lactic acid) (PDLA-D120, *M*_w_ = 157 kg/mol, PDI = 1.99, further referred to as PDLA), were kindly provided by TotalEnergies Corbion (Gorinchem, The Netherlands). Moreover, a styrene-acrylonitrile-glycidyl methacrylate ternary random copolymer (SAN-*g*-GMA) (SAG002, with epoxy content of 2 wt % and *M*_w_ = 80 kg/mol, further referred to as SAG) was kindly provided by Fine-blend Polymer Co, Ltd. (Shanghai, China).

### Sample Preparation

The sample preparation involves an integrated grafting and blending process. First a reactive blending occurs, wherein SAG is subjected to interactions with PLLA or PDLA in the presence of PVDF, serving as the host matrix. This step aims at the formation of poly(styrene-*co*-glycidyl methacrylate)-*graft*-poly(l-lactic acid) (SAG-*g*-PLLA, further referred to as SAL) and poly(styrene-*co*-glycidyl methacrylate)-*graft*-poly(d-lactic acid) (SAG-*g*-PDLA, further referred to as SAD). The composition includes 7 wt % of PLLA or PDLA, along with 3 wt % of SAG, complemented by 90 wt % of PVDF. Subsequent to the successful completion of the reactive blending phase, a precisely measured quantity of PLLA is added to obtain PVDF-SAD/PLLA or PVDF-SAL/PLLA blends with compositions of 50/50 wt %.

This blending process is conducted using a twin-screw microcompounder (15 cc, DSM Xplore, The Netherlands) under nitrogen flow. All the polymer pellets are dried at 70 °C for 12 h. During reactive blending, executed at 200 °C with a rotation speed of 50 rpm for a duration of 5 min, PVDF, SAG, and the PLA enantiomers, i.e., PLLA or PDLA, are added sequentially. The subsequent melt blending phase is carried out at the same temperature and rotation speed, for an additional 5-min interval after feeding the PLLA. The same procedure for the reactive melt blending, involving SAG and PLA enantiomers (PDLA/SAG or PLLA/SAG 95/5), is applied to generate SAL and SAD in the absence of PVDF. This method is conducted in parallel with the compatibilizers produced in the presence of PVDF, for comparison purposes. Furthermore, the formulation of pure PVDF/PLLA blends at a ratio of 50/50 wt % is achieved by utilizing the conditions outlined in the second blending phase.

In the context of sample preparation for different characterizations, a precise amount of predried samples is subjected to compression molding to make circular disks with dimensions of 25 mm in diameter and 500 μm in thickness. This is achieved using a compression machine (Fontijne Holland TP400). During this process, the specimens are positioned between aluminum plates of 2 mm thickness. To mitigate adhesion issues, the plates are covered with aluminum sheets, each having a thickness of 0.2 mm. The entire assembly is subsequently subjected to controlled temperature conditions at 200 °C, and compression is maintained at a force of 50 kN for a duration of 2 min. After that, the stack is quenched in an ice–water bath. For the DSC measurements, circular disks with a diameter of 4 mm are punched from the compression-molded disks. Similarly, an analogous methodology is applied to fabricate disks with a thickness of 1 mm, specifically for X-ray diffraction (XRD) and rheometry. Additionally, the same procedure is adopted to produce sheets with a 500 μm thickness for mechanical characterization purposes. Tensile samples with a cross-section of 5 × 0.5 mm^2^ are cut from the compression-molded sheets, according to the ISO527-1BA geometry standard. The sheets are punched into a rectangular shape of 30 × 7 × 0.5 mm for DMTA analysis. Sliced samples are also prepared using a Leica RM2165 microtome equipped with a Cryo-unit Leica LN21 for nano-IR measurements. The samples are cut from the cross-section of the disks using a diamond knife at −20 °C and mounted in a special AFM holder.

### Characterization

#### Infrared Spectroscopy

Fourier-transform infrared (FTIR) spectroscopy measurements are conducted in transmission mode using a Nicolet iN10 infrared spectrometer using the internal Globar infrared source at Helmholtz-Zentrum Berlin, with a resolution of 2 cm^–1^ by averaging 32 scans between 4000 cm^–1^ and 600 cm^–1^. For each sample, a thin film of approximately 5 μm thickness is fixed with a holder, and the measurements are performed at room temperature.

#### Wide-Angle X-Ray Diffraction

The crystalline phase of the prepared samples is assessed using a wide-angle X-ray diffraction (WAXD) setup situated at the BL11 NCD-SWEET beamline of the ALBA synchrotron in Barcelona, Spain.^38^ Quasi-2D WAXD patterns are captured by an LX255-HS area detector with a pixel size of 44.3 × 44.3 μm^2^ (Rayonix, Evanston, Illinois) positioned 169 mm away with a tilt angle of 30°. The beamline operates at a photon energy of 12.4 keV, corresponding to a wavelength (λ) of 0.999 Å. Calibration for WAXD is performed using chromium(III) oxide (Cr_2_O_3_). Samples are affixed to an aluminum multisample holder with 4 mm holes using double-sided adhesive tape, and measurements are made at room temperature. For all diffraction measurements, the obtained patterns undergo normalization to the incident beam intensity and background subtraction. Data integration is carried out using the BUBBLE open-source software, provided by the European Synchrotron Radiation Facility (ESRF).^39^

#### Differential Scanning Calorimetry

A Mettler Toledo DSC823e instrument is utilized for conducting differential scanning calorimetry (DSC) measurements. Specimens, weighing approximately 7–9 mg, are obtained from hot-pressed samples and sealed using aluminum pans for analysis. During DSC testing, a continuous nitrogen purge of 50 mL/min is maintained to minimize potential thermal degradation throughout the measurements. The crystallization and melting behaviors are followed through nonisothermal crystallization processes. This involves an initial heating phase at 10 °C/min, wherein the samples are heated from room temperature to 200 or 250 °C, succeeded by a 2 min annealing interval at each of the mentioned temperatures. Subsequently, the samples undergo cooling to 25 °C at a rate of 10 °C/min, followed by reheating to 250 °C using the same heating rate for an assessment of their melting behavior.

#### Rheological Characterization

The linear viscoelastic behavior of the blends is investigated using an Anton Paar rotational rheometer (MCR 502) equipped with a convection oven and parallel plate geometry (diameter of 25 mm and gap size of approximately 1 mm). Strain sweep tests are carried out at a frequency of 1 rad/s (γ = 0.01%–1000%) at 200 °C. Using this temperature, small amplitude oscillatory frequency sweeps are performed at γ = 1%, spanning angular frequencies ranging from 0.05 to 600 rad/s. To evaluate the effect of stereocomplexation on the measurements, the procedure is replicated after subjecting the samples to melting at 250 °C. In that case, in the initial phase, the samples are positioned between the plates at 200 °C, followed by thermal annealing at 250 °C for 2 min to remove any potential residual SC crystals. The gap size is subsequently adjusted to achieve an approximate gap height of 1 mm, and the sample is cooled at a rate of −10 °C/min until attaining the measurement temperature (200 °C). To mitigate oxidation effects, each experiment is executed with freshly prepared samples under a nitrogen-controlled atmosphere.

#### Infrared Nanospectroscopy and Imaging

IR nanospectroscopy and imaging experiments are done via the scattering-type scanning optical microscopy technique. The measurements were done at the IR-nanospectroscopy end-station, IRIS beamline, BESSY II synchrotron, using an s-SNOM setup (neaScope, Attocube, Haar, Germany) coupled to brilliant broadband synchrotron infrared light.^40^ The measurements are performed in the tapping mode using Pt–Ir-coated AFM-probes with a typical tip radius of <25 nm (Arrow NCPt, NanoWorld, Neuchâtel, Switzerland), The cantilever is driven close to its resonance oscillation frequency of ∼255 kHz and with a contact tapping amplitude of ∼75 nm. The rather high oscillation amplitude used in the experiment implies tip–sample contact and dominant repulsive interactions at one end and attractive forces at the other end of the oscillation. The imaging is performed in the zero path difference heterodyne imaging or “white light” imaging mode using broadband infrared synchrotron radiation.^41^ For the acquisition of local nano-IR spectra, the AFM tip is fixed at a specific location and the nano-FTIR spectra are collected in the range of 600–2100 cm^–1^ with a nominal spectral resolution of 8 cm^–1^, and each spectrum is an average of 20 interferometric scans. The collected spectra are referenced to the spectrum of Si, recorded using the same AFM-probe and under the same experimental conditions. The conversion of the recorded interferograms to the infrared amplitude and phase spectra is done using a script developed in-house at the IRIS beamline in the SciLab open-source software using an asymmetric apodization window based on the three-term Blackman-Harris function and with a zero-filling factor of 4. The reference-corrected amplitude and phase signals are used to calculate the nano-FTIR absorption = amplitude*sin(phase).^40^ For both the imaging and the spectroscopy modes, the detector signal is demodulated at the second harmonic of the AFM oscillation frequency, and the corresponding optical amplitude and phase signals are used to obtain the images and spectra shown in the present work. Use of the heterodyne detection mode and the second demodulation harmonic allows to isolate the near-field tip–sample interaction signal.^41^ In addition to the optical images, the setup allows simultaneous collection of the AFM images (topology, mechanical phase, and amplitude signals), which were also used for characterization of the blend materials. All measurements are performed at room temperature in a nitrogen atmosphere.

#### Mechanical Analysis

Uniaxial tensile tests are carried out using a Zwick Z010 universal tensile testing machine, which is equipped with a 10 kN load cell. The tests are conducted at a constant strain rate of 10^–3^ s^–1^ at room temperature. For statistical purposes, each condition is tested at least in triplex, and the specimen with the highest strain at break for each sample is reported in the comparative figure. Dynamic mechanical thermal analysis (DMTA) is performed using a Hybrid Rheometer HR30 (HR30-TA Instruments) equipped with a 50 N axial load cell. The analysis includes a heating rate of 3 °C/min, a temperature ranging from −70 to 150 °C, a constant axial displacement of 15 μm, and a frequency of 1 Hz under nitrogen flushing at 10 mL/min. Quasi-static fracture tests (crosshead speed: 5 mm/min) are performed on single-edge notch bending (SENB) specimens. The samples with dimensions of 55.5 × 5.3 × 4.8 mm^3^ are prepared using compression molding at 200 °C. An initial notch of 1.5 mm in length is made with a saw. A sharp precrack, approximately 0.25–0.3 mm in length, is created at the end of the sawed notch by tapping with a razor blade, resulting in an initial notch length close to 1.8 mm. From these experiments, the fracture toughness K_IC_ was determined according to ASTM E1820.

#### Scanning Electron Microscopy (SEM)

Scanning electron microscopy (SEM) images are obtained using an SEM (Thermofisher Quanta 600F) at an acceleration voltage of 10 kV. To prepare the specimens for SEM observations, they are cut from the surfaces of the samples after the quasi-static fracture tests and then sputter-coated with a thin layer of gold in a vacuum chamber.

## Results and Discussion

### Formation of Reactive Compatibilizers

**Figure 1:**
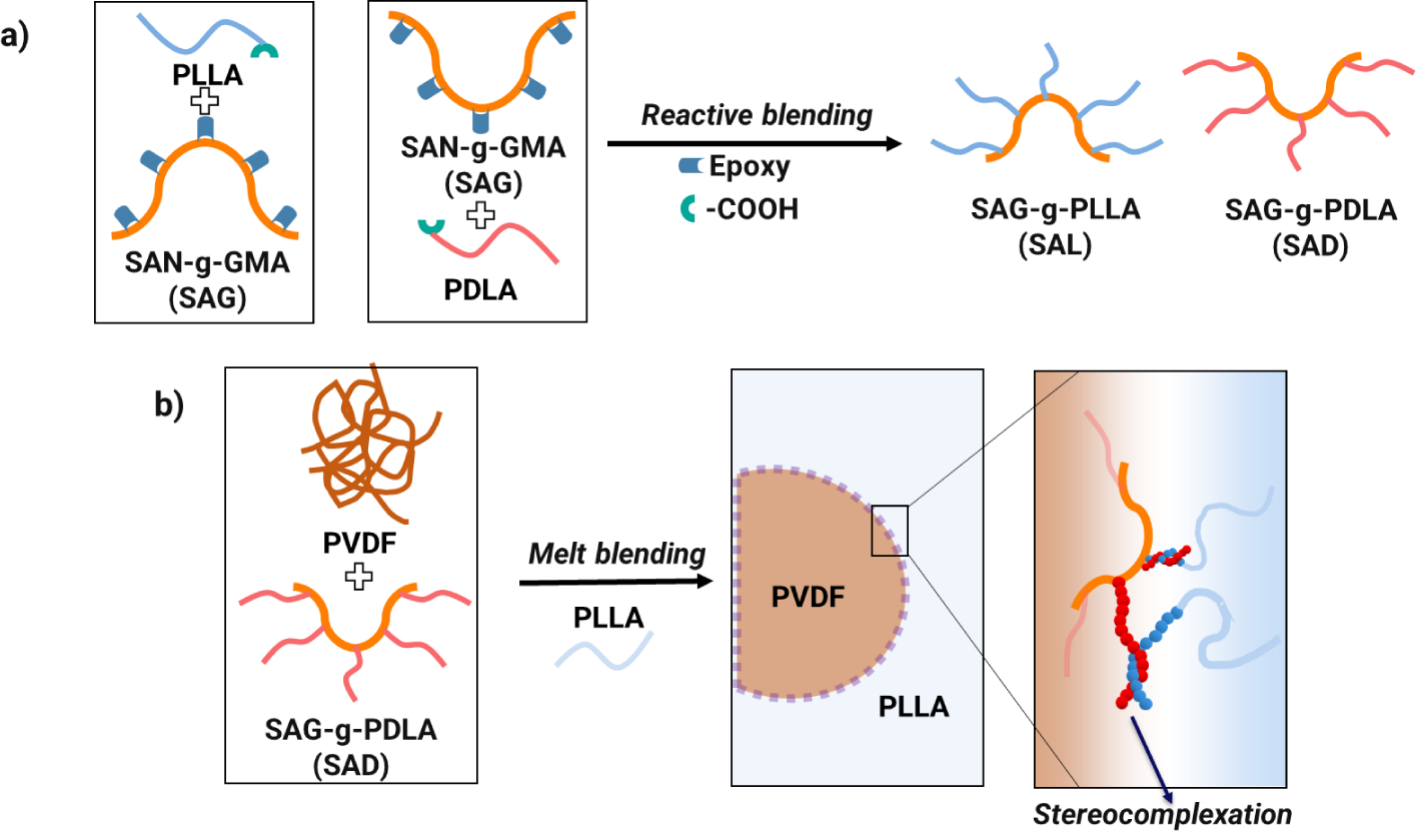
Schematic illustrating: a) the formation of SAG-*g*-PLLA (SAL) and SAG-*g*-PDLA (SAD) copolymers during reactive blending. b) The process of crystal formation at the interface of a PVDF/PLLA blend.

During the melt blending process, the epoxy group present in glycidyl methacrylate within SAN-*g*-GMA (SAG) can undergo an in situ reaction with the carboxyl and hydroxyl terminal groups of PLLA and PDLA (Figure 1). This chemical interaction leads to the formation of SAG-*g*-PLLA (SAL) and SAG-*g*-PDLA (SAD), which subsequently serves as a potential compatibilizer for the PVDF/PLLA blend. As described in the “Sample Preparation” section, this reaction transpires within the PVDF phase. It has been observed that the styrene-acrylonitrile (SAN) phase demonstrates miscibility with PVDF at this composition;^42,43^ the procedure is thus anticipated to yield a homogeneous mixture of PVDF and SAG. In addition, due to the presence of epoxy groups randomly dispersed along the main chains of SAG, the reactive blending process allows for the grafting of PDLA and PLLA onto SAG. This grafting occurs through ring-opening reactions involving the epoxy group in the GMA part of SAG and the carboxyl groups of PLA enantiomers during the melt blending process. However, to understand the interaction dynamics between SAG and the PLA enantiomers, PDLA and SAG are also blended in a 95/5 (w/w) ratio, in the absence of PVDF.

**Figure 2:**
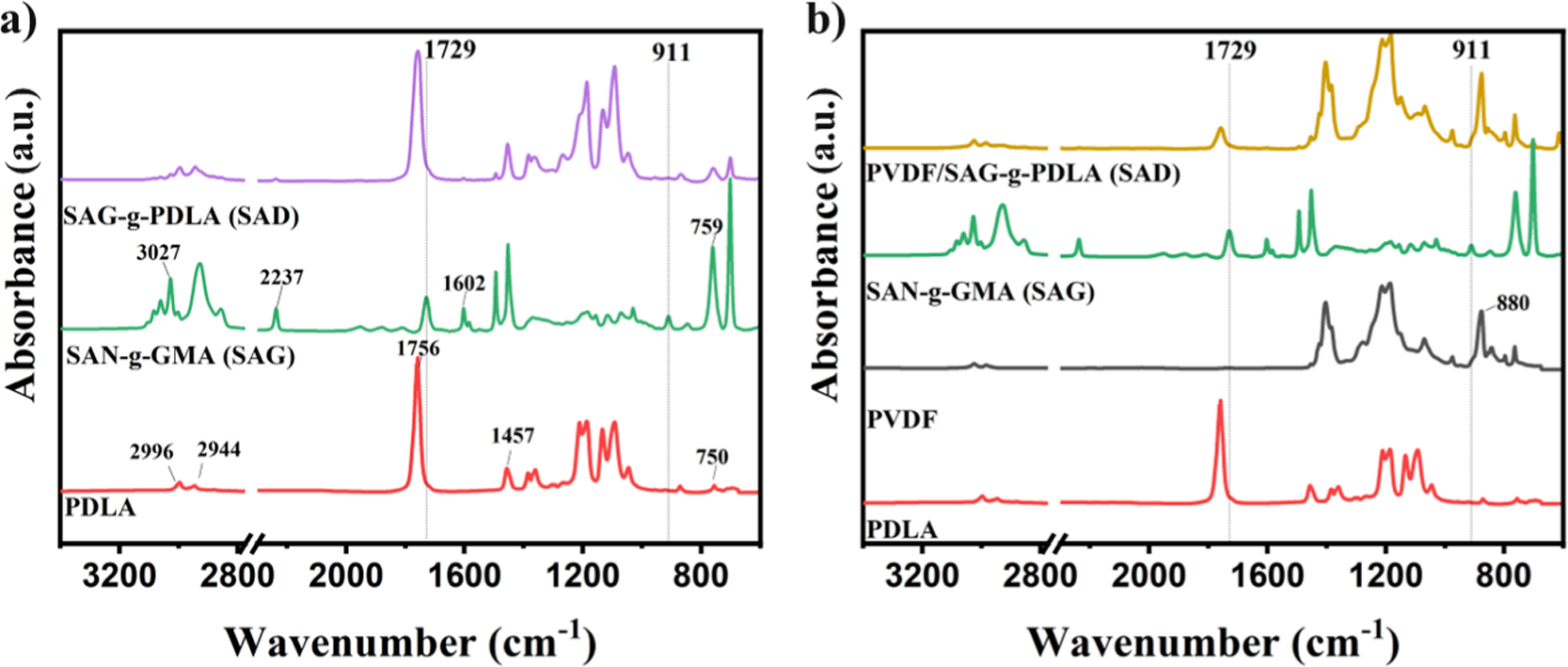
FTIR spectra of PDLA, SAN-*g*-GMA (SAG), and SAG-*g*-PDLA (SAD) formed within a) PDLA or b) PVDF phases.

The formed SAG-*g*-PDLA is characterized using FTIR, and the results are compared with those of compatibilizers prepared inside the PVDF phase in Figure 2a,b. For the pure PDLA, the absorption peaks observed at 750 cm^–1^, 1457 cm^–1^, and 1756 cm^–1^ correspond to the C=O bending, the deformation of C–H bonds, and the stretching vibration of C=O bonds, respectively.^44^ Additionally, the absorption peaks detected at 2996 cm^–1^ and 2944 cm^–1^ are attributed to the presence of C–H bonds within the aliphatic group.^44,45^ For the SAG copolymer, the peaks at 3027 cm^–1^ and 1602 cm^–1^ can be ascribed to the stretching vibrations of C–H bonds, C=C bonds, and the styrene ring, respectively.^46^ Additionally, an absorption peak located at 759 cm^–1^ corresponds to the out-of-plane bending vibration of C–H bonds within the styrene ring, and the presence of a peak at 2237 cm^–1^ is attributed to the stretching of C≡N triple bonds.^46,47^ In parallel, glycidyl methacrylate (GMA) exhibits distinct peaks at 911 cm^–1^ and 1729 cm^–1^ which are indicative of the presence of epoxy groups and carbonyl groups (C=O), respectively.^48,49^

Following the reaction between GMA and PLA enantiomers, two prominent changes become evident. Specifically, a decrease in the intensity of the epoxy characteristic peak at 911 cm^–1^ is observed after the blending of SAG and PDLA (Figures 2a and S1). This observation implies the occurrence of reactions involving the epoxide groups with the −COOH and −OH groups, concluding in the formation of SAG-*g*-PDLA.^49^ Furthermore, a second change pertains to the merging of the characteristic peaks at 1756 and 1729 cm^–1^. These peaks are attributed to the stretching vibration of the carbonyl group in PDLA and GMA, respectively. This merging occurs within the spectrum of SAG-*g*-PDLA, suggesting the grafting of PDLA and PLLA onto the chains of SAG.^10^ FTIR spectra of SAG-*g*-PLLA (SAL) can be seen in Figure S2, showing similar trends.

A similar trend is evident in the case of SAG-*g*-PDLA synthesized in the presence of PVDF, as shown in Figure 2b. However, due to the coinciding characteristic peak of PVDF near 880 cm^–1^, which corresponds to the CF–CH–CF bending vibration,^50,51^ the reduction in the 911 cm^–1^ peak is less discernible. Nevertheless, the overlap of the peaks at 1756 and 1729 cm^–1^ remains distinct in the FTIR results (Figures 2b and S3). This observation reveals that the grafting reaction between the PLA enantiomers and SAG can occur effectively within the PVDF phase. It has been documented that the binary blend of PVDF/SAN constitutes a partially miscible system.^42,43^ Upon analyzing the structural composition of SAN and PVDF, it becomes apparent that both polymers possess polar attributes. SAN contains a robustly polar −CN group, while PVDF features two fluorine atoms. This composition hints at the existence of specific interactions between the dipole in PVDF and the nitrile group in SAN. Furthermore, the incorporation of the methacrylate group within the GMA component enhances the miscibility of the SAG/PVDF system.^52^ Consequently, it can be anticipated that SAG is uniformly distributed within the PVDF phase. This uniform distribution would facilitate the reaction between the SAG epoxy groups and the COOH groups of PDLA upon the addition of PDLA to the system.

NMR measurements confirm the successful reaction between PDLA and SAN-GMA. This is evidenced by a peak disappearance observed in the ^1^H NMR spectra within the signal corresponding to the methine proton at the α-position relative to the terminal −OH group of PDLA, around 4.4 ppm, indicating the substitution of PDLA’s terminal hydroxyl groups with GMA.^53^ This is shown in Figure S4a. This peak disappearance is indicative of the grafting process, providing strong evidence of the formation of SAG-*g*-PDLA.

### Morphological Characterization and Visualization of SC crystals’ Location

As described in the previous section, the formation of SAD and SAL, i.e., SAG-*g*-PDLA or SAG-*g*-PLLA, copolymers, respectively, takes place within the PVDF phase. Upon the addition of PLLA to this blend, these copolymers exhibit potential compatibilizing characteristics for PVDF/PLLA 50/50 immiscible blends. Particularly, the SAN component within SAG displays complete incompatibility with the PLLA phase. Consequently, following the migration of SAD or SAL toward the PVDF/PLLA interface, the SAN segment would predominantly reside within the PVDF phase, while the PDLA or PLLA segments would predominantly localize within the PLLA phase. Consequently, SC crystals can form via paired helices formation between l and d enantiomers in the PVDF/SAD/PLLA blend, whereas only PLLA chain entanglement is expected in the PVDF/SAL/PLLA blend.

**Figure 3:**
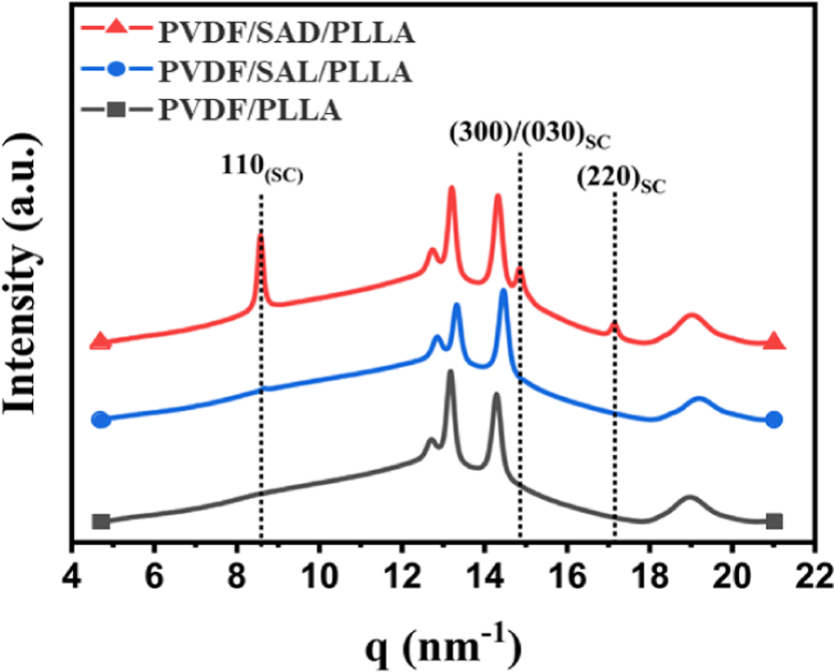
WAXD profiles of the pure PVDF/PLLA blend and blends with various compatibilizers.

X-ray diffraction (XRD) is used to validate the presence of SC crystals within the resultant blends. WAXD measurements are employed to compare the crystalline structure of the samples and to quantify the precise amount of SC crystals in the PVDF/SAD/PLLA blend. Figure 3 reveals three distinct characteristic peaks at around 8, 14, and 17 nm^–1^ in the WAXD patterns, corresponding to the (110), (300)/(030), and (220) planes of the SC crystals, respectively.^54^ These peaks are exclusively evident in the blend compatibilized with SAD. It should be noted that the additional peaks belong to the PVDF crystalline phase, emerging due to its rapid crystallization kinetics during the cooling process after pressing.

**Figure 4:**
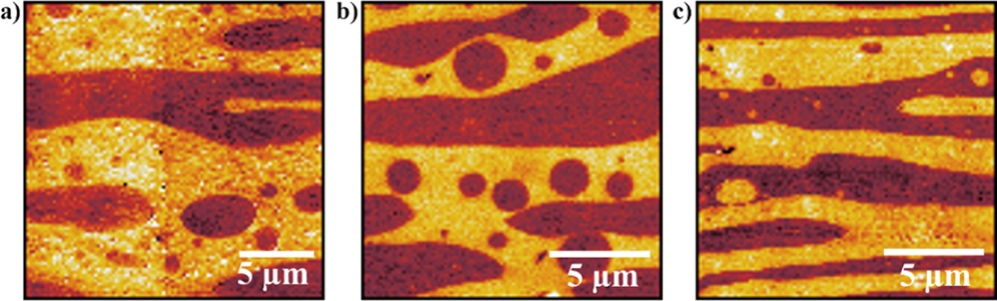
Nano-IR optical amplitude images of a) pure PVDF/PLLA blend, b) with SAL compatibilizer (PVDF/SAL/PLLA), and c) with SAD compatibilizer (PVDF/SAD/PLLA). The light and dark colors correspond to the PLLA and PVDF phases, respectively.

The introduction of SAD or SAL into the system and the ICIC effect can cause alterations in the morphology type and phase dimensions. To investigate the compatibilization effect, the morphology of both pure PVDF/PLLA blends and blends incorporating SAD or SAL are subjected to comprehensive characterization using the infrared s-SNOM technique. Figure 4 shows the optical amplitude images collected with the s-SNOM technique. Strong contrast in the optical signal is observed between the different polymers present in the blends. The images indicate that all of the 50/50 blends exhibit a cocontinuous morphology, which also can be seen in the AFM images in Figure S5. Moreover, they suggest a slight reduction of the characteristic domain sizes in the PVDF/SAD/PLLA system compared to the neat PVDF/PLLA system and the PVDF/SAL/PLLA system. In the former blend, SAD can have a dual influence namely the copolymer compatibilization capability coupled with the presence of a solid interface engendered by stereocomplexation. SC crystallization at the interface allows to bridge the polymer phases, thereby stabilizing the transient cocontinuous structures that arise during the melt mixing process. These effects can result in a finer and more uniformly distributed domain arrangement, as has been observed earlier by Wang et al. in nanoparticle-filled PVDF/PLLA blends.^55^

The presence of nanoparticles localized at the blend interface leads to morphology refinement due to the particles’ compatibilization effect and their influence on interfacial rheology.^6,7,56^ Specifically, this results in delayed relaxation of elongated phases and reduced interfacial energy.^6,56^ For ICIC via stereocomplexation, the effectiveness of compatibilization intrinsically relies on localization of the crystals, similar to what is observed in nanoparticle-induced compatibilization. To elucidate the precise location of the stereocomplexation process in the PVDF/SAD/PLLA blend, the infrared nanospectroscopy technique is used to study the PVDF/SAD/PLLA interface.

**Figure 5:**
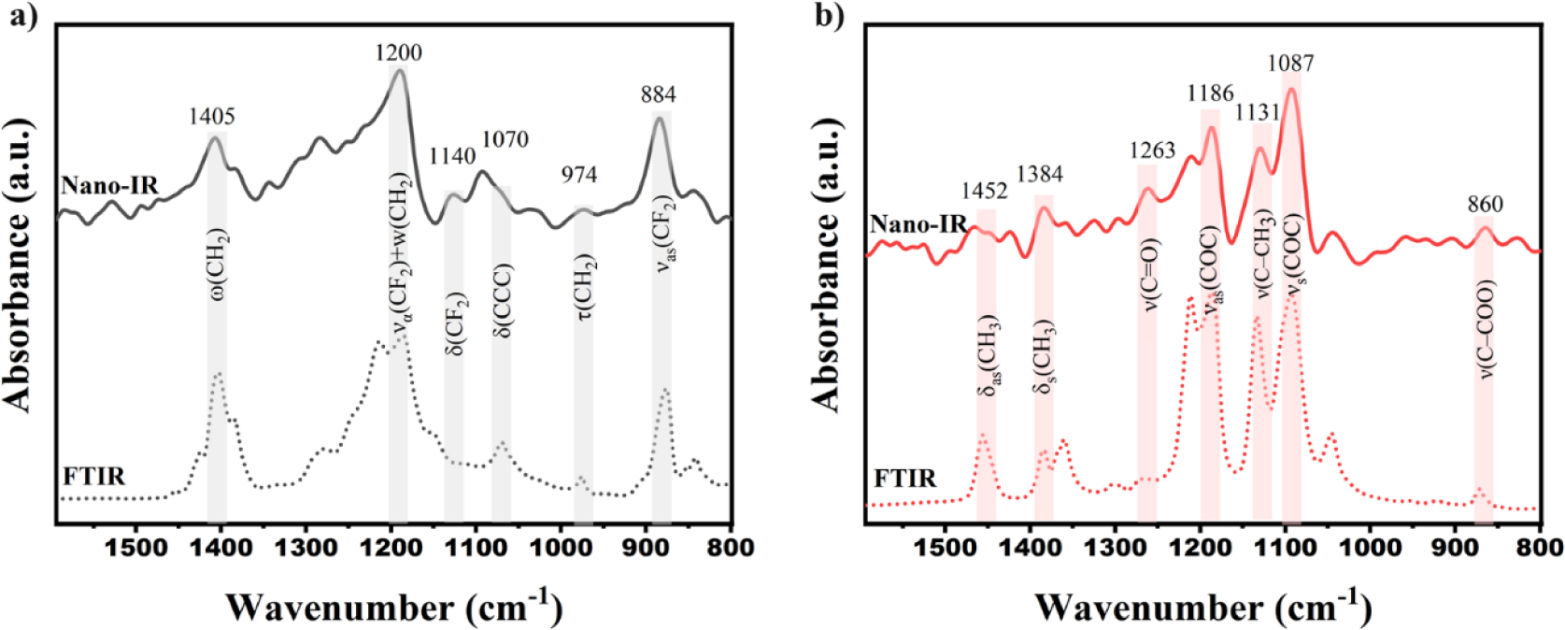
Comparison of nano-IR (solid line) and FTIR spectra(dotted line) in the range 1600–800 cm^–1^ for a) PVDF and b) PLLA. *δ;* bending,*v;* stretchingvibration, *w;* wagging, *τ;* twisting.

**Figure 6:**
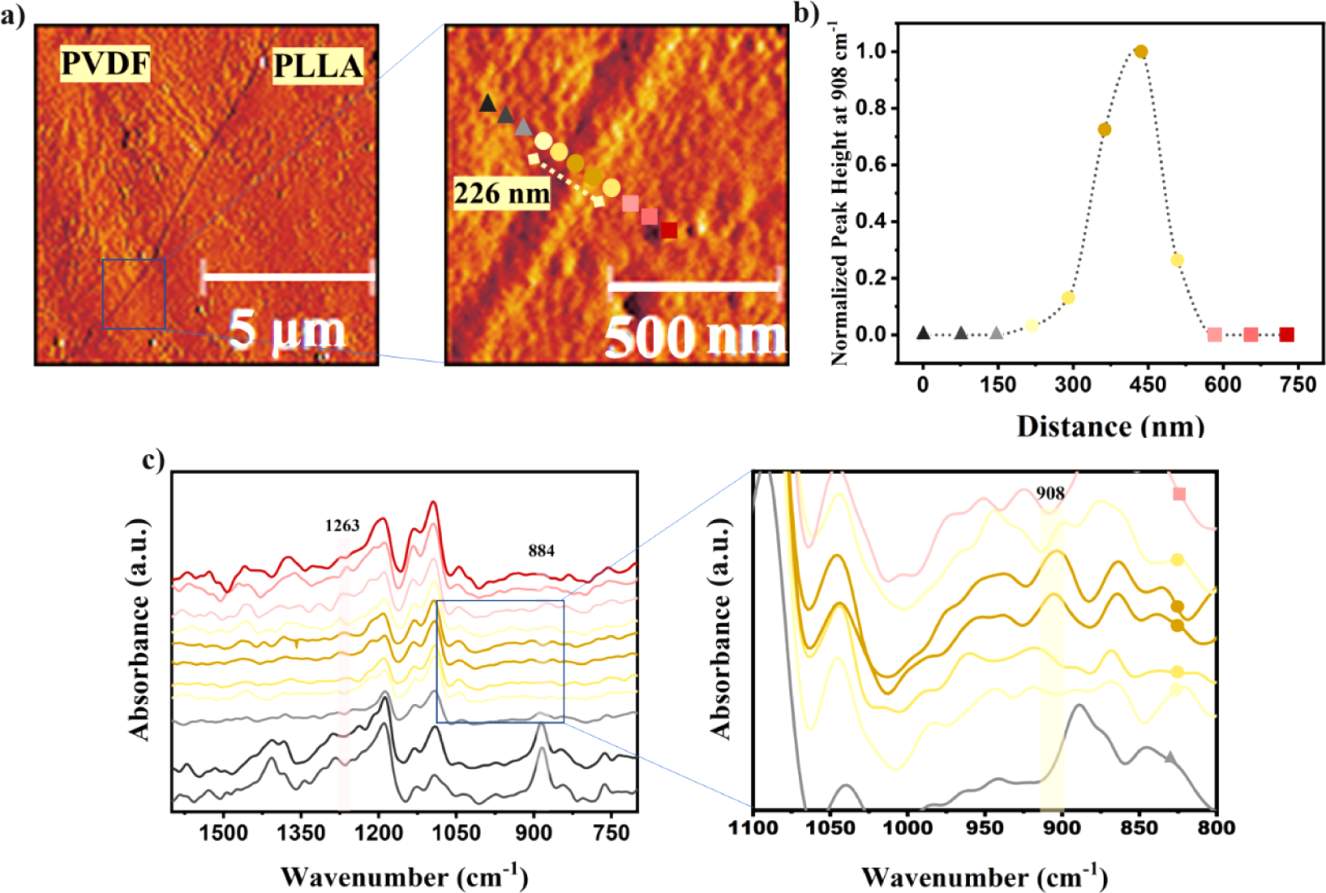
(a) AFM mechanical phase images captured at different scales, showing the PVDF/SAD/PLLA blend interface. (b) Normalized peak height obtained from the characteristic peak of the SC crystals at 908 cm^–1^ illustrating the thickness of the interface layer; the normalization is done by the maximum intensity value at this wavenumber; triangles, circles, and rectangles represent the PVDF phase, interface, and PLLA phase, respectively; the dotted line is a guide-to-the-eye. (c) Nano-IR spectra recorded from regions indicated by the colored spots in the AFM image, spaced at 70 nm, with a zoom of the spectral region spanning 1100–800 cm^–1^. The spectra corresponding to PVDF are depicted in black, those from PLLA are in red, and those corresponding to the interface are in yellow. The colors of the spots correspond to the respective spectra colors.

Figure 5 displays the FTIR spectra of pure PLLA and PVDF, along with nano-IR spectra collected from the PVDF and PLLA phases of the blend. The assigned IR bands presented in Figure 5a,b correspond to characteristic infrared bands of PLLA and PVDF as collected from previous studies.^44,51,57^ Indeed the simple interpretation of the complex near-field signal collected via the s-SNOM technique used in this work is a mathematical simplification, which allows to get spectra comparable to the far-field infrared spectroscopy techniques in case of weak oscillators.^36^ The precise positions of the peaks may vary slightly between the methods based on factors such as sample crystallinity and measurement conditions. Moreover, precise interpretation of the collected near-field spectra data requires modeling of the interaction between the AFM-tip^58^ and the sample, which is out of scope of this article. The agreement observed between the nano-IR results and conventional FTIR data affirms the validity of the nano-IR measurements, as evidenced by the nearly identical spectra of PLLA obtained through both methods. In the case of PVDF, the main bands can be seen in the spectra collected using both methods; however, the relative intensity of the bands is different. From the AFM mechanical phase images (Figure 6a), it is seen that, in the PVDF phase, spherulite crystals can be present in the plane of the sample cut. It is known that the s-SNOM technique is more sensitive to the out-of-plane vibrational bands,^59^ and presence of the preferentially orientated PVDF crystals can explain the different relative intensities of the vibrational bands in the nano-IR spectrum.

Figure 6a shows the AFM mechanical phase images of the PVDF/SAD/PLLA cross sections. In the AFM mechanical phase images, the interface between PVDF and PLLA reveals a well-defined layer with an approximate thickness of roughly 230 nm. Figure 6c shows the nano-IR spectra recorded across the interface and in each phase of the PVDF/SAD/PLLA blend. The regions with colored spots in the enlarged AFM image (Figure 6a), spaced at 70 nm intervals, mark the locations where the nano-IR spectra are collected. One can see the changes in the acquired spectra upon crossing the interface whereby the bands corresponding to one phase gradually disappear, while other vibrations start to be dominant. For instance, the well-defined peak associated with the CF_2_ stretching at 884 cm^–1^ in PVDF fades near the interface. Simultaneously, the presence of C==O vibrations from PLLA becomes noticeable, particularly at 1263 cm^–1^ at the last data point.

The s-SNOM results, both AFM images and the nano-IR spectra, in the interface layer region clearly reveal the presence of a thin layer that cannot be ascribed to PVDF or PLLA phases. The nano-IR results distinctly show the presence of a peak at 908 cm^–1^ corresponding to the characteristic IR absorption of SC crystals.^54^ The boundary of the interface layer extending toward the PLLA and PVDF phases is defined by the absence of this peak. Figure 6b illustrates variations in SC concentration at the interface by plotting the peak heights at 908 cm^–1^ for each point, spaced at a distance of 70 nm and normalizing them to the maximum peak height observed in this region. Based on this analysis, the thickness of the SC layer at the interface in this specific region of the blend is approximately 250 nm, which agrees well with the estimation done using the AFM mechanical phase image (Figure 6a). The good agreement between values of the interface layer thickness obtained from the AFM mechanical phase image and the nano-FTIR line scan confirms that the band at 908 cm^–1^ is characteristic of the SC phase and that the nano-FTIR technique is capable of detecting this spectral feature against the background of other intense spectral bands and the overall signal noise level.

The collected AFM and nano-IR spectroscopy results at the nanoscale support the formation of SC crystals through interchain interactions involving the PDLA portion of the SAD copolymer and the PLLA chains at the blend interface. Previous studies have hypothesized the presence of SC crystals at the interface of polymer blends, with their existence being supported by indirect methods such as rheological techniques.^9−12^ The near-field infrared nanospectroscopy technique used in this work for the first time, to the best of our knowledge, provides direct evidence of the formation of SC crystals at the polymer blend interface. This SC crystalline layer is expected to enhance the work of adhesion between the blend phases and facilitate more favorable interdiffusion within the PLLA and PVDF blend. As a result, it is expected to lead to an improved thermo-mechanical performance of the blend.

### Effect of Stereocomplexation at the Interface on Thermal and Rheological Properties

In the previous section, it was shown that SC crystals exist at the interface of a PVDF/SAD/PLLA blend. Although the addition of the compatibilizer preserves the cocontinuous structure (Figure 4), it changes the interface thickness, which is expected to affect the viscoelastic and crystallization behavior of the system. In this section, effect of the SAL and SAD compatibilizers at the interface, whereby the latter involves ICIC on the thermal and rheological behavior of the PVDF/PLLA blend is examined.

**Figure 7:**
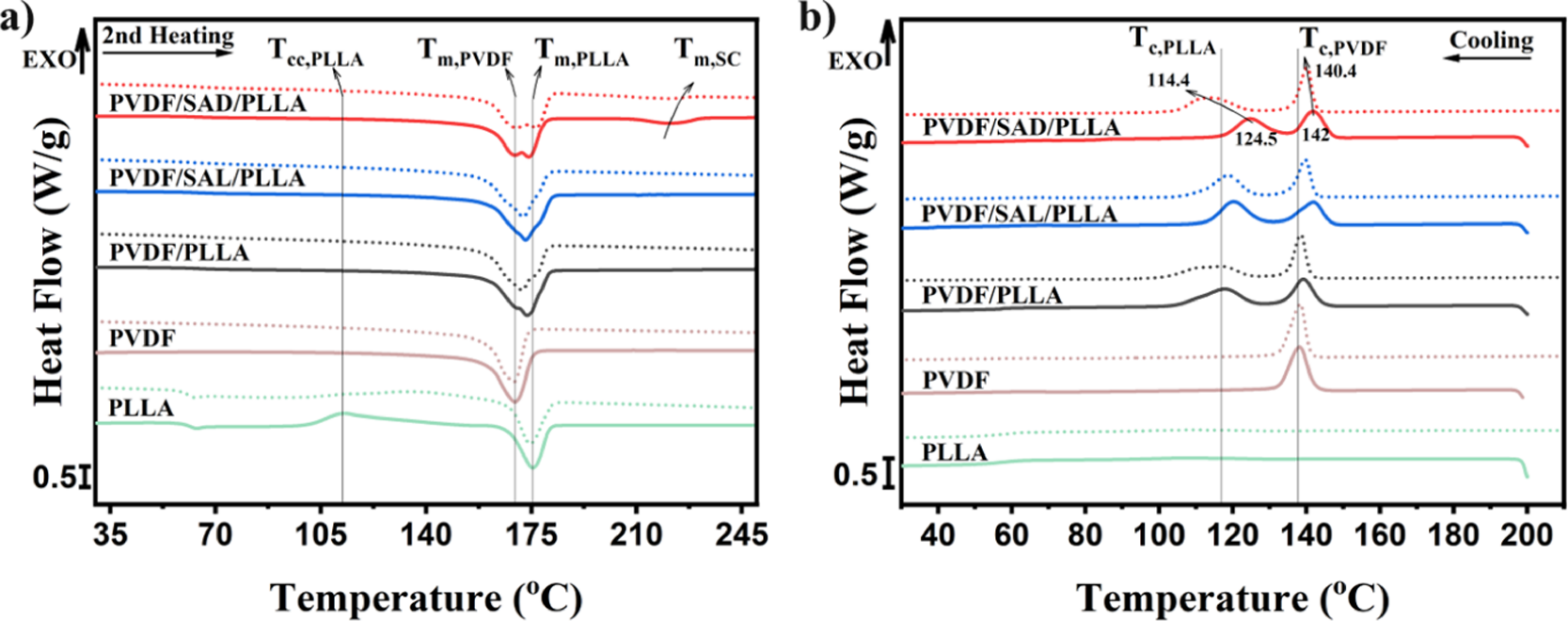
Effect of compatibilizers and stereocomplexation on nonisothermal crystallization behavior during a) second heating from room temperature after cooling temperatures of 200 °C (solid line) and 250 °C (dotted line) and b) cooling from temperatures of 200 °C (solid line) and 250 °C (dotted line).

In immiscible blends, the crystallization behavior within their components becomes more intricate as compared to homopolymers. This complexity stems from the mutual influence exerted by coexisting phases. The crystallization process in polymer blends is governed by multiple variables, including domain size, nature and concentration of the compatibilizer, and presence of nucleating agents. Importantly, the phenomenon of nucleation at interfaces can play a pivotal role in the crystallization process in polymer blends.^21^ The introduction of a compatibilizer can decrease the domain size thereby increasing the interfacial area, and enhancing the nucleation rate. These effects can be analyzed by studying the nonisothermal crystallization behavior. The effect of SAD and SAL compatibilizers on the blend’s melting behavior and nonisothermal crystallization are probed by DSC measurements. Figure 7a reveals the melting behavior resulting from the second heating cycle initiated at room temperature after cooling from 200 or 250 °C. The melting points corresponding to the homocrystals of the PLLA and PVDF phases are observed to occur within the temperature range of 160 to 180 °C. This consistent trend can be seen for all the blends. However, an additional melting peak appears within the range of 205 to 235 °C exclusively for the PVDF/SAD/PLLA blend, characterizing the melting of SC crystals.

Comparing the crystallization behavior of the components within the blend with that in their pure state after cooling from 200 °C (Figure 7b) reveals that the crystallization temperature of PVDF and PLLA increases after blending and melt crystallization of PLLA occurs significantly in the blends. However, it is noteworthy to mention that no cold crystallization within the PLLA component is observed in any of the blend samples during the second heating (Figure 7a), which is different from pure PLLA. This observation serves to highlight the presence of heterogeneous nucleation phenomena caused by the blend interface.^60^ The consequential increase in the crystallization kinetics of PLLA becomes evident, and thus, the crystallization of the PLLA phase occurs effectively during the primary cooling process.

To uncover more perspective, an intricate comparison is made between the nonisothermal crystallization behaviors, subsequent to cooling from temperatures of 200 and 250 °C. The selection of these thermal regimes strategically corresponds to scenarios where SC crystals remain in their solidified and molten state, respectively. As depicted in Figure 7b, during the cooling process from the elevated temperatures, two exothermic peaks become evident. These peaks correspond to the crystallization temperatures of PVDF and PLLA (*T*_c,PVDF_ and *T*_c,PLLA_), as illustrated in the plot. The *T*_c,PVDF_ and *T*_c,PLLA_ values after cooling from 200 and 250 °C are presented in Table 1. Comparing the *T*_c_ values of PLLA and PVDF in the pure and compatibilized blends obtained during cooling from 200 °C reveals that the incorporation of SAD and SAL compatibilizers into the PVDF/PLLA blend leads to higher crystallization temperatures of both PVDF and PLLA. At the used cooling rate, the SC crystals persist within the PVDF/SAD/PLLA blend, resulting in noticeably higher *T*_c_ values compared to the other samples. These enhanced crystallization temperatures can be attributed to the heterogeneous nucleation effect,^60^ which can be fortified by the nucleation ability of the SC crystals for PLA homocrystallization at the interface as compared to amorphous chains.^21^ In addition, the slight increase in interfacial area for the PVDF/SAD/PLLA blend as compared to the other blends increases this effect even more. Although the difference in *T*_c,PVDF_ is not substantial, the relatively higher *T*_c_ values for PVDF/SAD/PLLA and PVDF/SAL/PLLA compared to the pure PVDF/PLLA blend can be attributed to the nucleating effect of the interface and the presence of impurities, such as unreacted components within the PVDF matrix.

**Table 1:** Crystallization Temperature of PVDF (*T*_c,PVDF_) and PLLA (*T*_c,PLLA_) in the Pure and Compatibilized PVDF/PLLA Blends After Cooling from 200 and 250 °C

	Cooling from 200 °C		Cooling from 250 °C	
Sample name	*T*_c,PLLA_ (°C)	*T*_c,PVDF_ (°C)	*T*_c,PLLA_ (°C)	*T*_c,PVDF_ (°C)
PVDF/PLLA	117.6	139.1	115.9	138.9
PVDF/SAL/PLLA	119.8	141.8	118.5	139.6
PVDF/SAD/PLLA	124.5	142	114.4	140.4
PLLA	108.4	-	109.1	-
PVDF	-	138.1	-	137.7

The crystallization behavior of the blend components in the pure and compatibilized blends during cooling from 200 °C is also compared with the crystallization behavior of the components after cooling from 250 °C where the impact of stereocomplexation becomes more evident because the effect of SC crystals is eliminated by melting them. As can be seen in Table 1, the *T*_cPLLA_ decreases approximately 10 °C from 124.5 to 114.4 °C after melting the SC crystals. However, the reduction in *T*_c,PVDF_ under these conditions is less significant. This observation provides clear evidence of the influence of the SC crystalline layer on the nucleation of the PLLA phase within the blend. A slight decrease in *T*_c,PVDF_, and *T*_c,PLLA_ is also observed for both PVDF/SAL/PLLA and PVDF/PLLA blends following cooling from 250 °C compared to cooling from 200 °C. At the higher temperature, the phase viscosity decreases significantly, and the morphology can coarsen rapidly due to the interfacial tension and curvature effects.^62,63^ Consequently, the interfacial area between the phases can decrease, thereby weakening the nucleating effect of the interface on the crystallization temperature. It should be noted that investigating the crystallization behavior of the pure components cooled from both 200 °C and 250° shows that no melt memory effect plays a role, and the effects observed in the neat and SAL-compatibilized blends are attributed to the heterogeneous nucleation effect of the formed interface within the blends.

**Figure 8:**
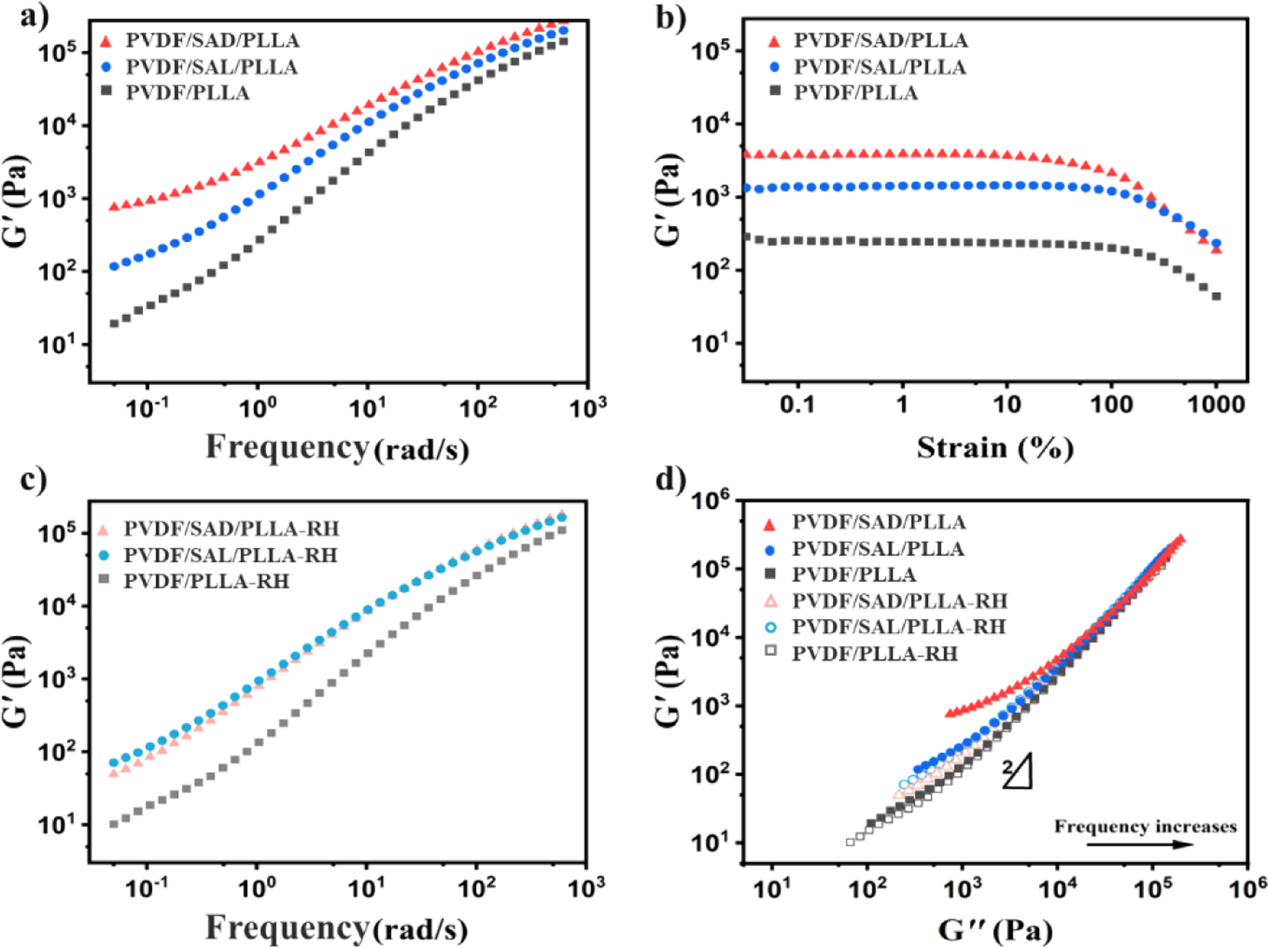
Rheological behavior of pure and compatibilized PVDF/PLLA blends at 200 °C, a) storage modulus *G*′ as a function of frequency, b) storage modulus *G*′ versus strain at frequency of 1 rad/s, c) storage modulus *G*′ as a function of frequency after reheating to 250 °C (abbreviated as RH on the plot), and d) storage modulus *G*′ as a function of loss modulus (Han plots) of the blends before and after reheating to 250 °C (abbreviated as RH on the plot).

As mentioned earlier, alterations in the interface state within the blends result from the presence of compatibilizers and the existence of SC crystals. These changes significantly influence the viscoelastic behavior of the blends. Figure 8a depicts the storage modulus as a function of the angular frequency measured at 200 °C for the pure PVDF/PLLA blend and the compatibilized blends. Evidently, the storage modulus of the pure blend exhibits a nonterminal behavior at low frequencies. This phenomenon arises from additional elasticity caused by the accumulated free energy at the interface of cocontinuous blends, which is contingent upon the interfacial area and curvature.^62,63^ Upon adding SAL to the PVDF/PLLA blend, the storage modulus increases, potentially due to the expanded interfacial area or minor cross-linking effects within the phases. The incorporation of SAD fosters the formation of SC crystals at the interface. This contributes an extra elastic effect, particularly at lower frequencies, indicating a more robust network structure due to the presence of SC crystals as they can form a physically cross-linked network in the system.^12,15^

Figure 8b illustrates the impact of shear strain on the dynamic storage modulus (*G*′) of the blends, with measurements conducted at 200 °C and a frequency of 1 rad/s. As can be seen, PVDF/SAD/PLLA exhibits a higher *G*′ value at low strain levels. Additionally, both PVDF/PLLA and PVDF/SAL/PLLA blends demonstrate linear behavior concerning *G*′ for strains up to nearly 100%. However, the *G*′ of PVDF/SAD/PLLA deviates from this linear behavior at approximately 10% strain. This transition highlights a distinctive sensitivity to shear strain exhibited by PVDF/SAD/PLLA blends, which results from their microstructure characterized by a robust interface and the additional networking effects of SC crystals, rendering it more susceptible to shear-induced network destruction.^64^ This implies that the rheological response of the PVDF/SAD/PLLA blend is dominated by the network structure of the SC crystals.

To gain more insight into the impact of the generated SC crystals on the rheological properties, the SAOS tests are also repeated following a two min annealing process at 250 °C, which causes the melting of existing SC crystals. Figure 8c illustrates the storage modulus as a function of frequency at 200 °C after annealing at 250 °C. A small decrease in the storage modulus (*G*′) subsequent to the 250 °C annealing is seen for all the blends. This can be attributed to the increased mobility of the system at this temperature, which accelerates the coarsening process of the cocontinuous morphology. Interestingly, the PVDF/SAD/PLLA and PVDF/SAL/PLLA blends exhibit indistinguishable rheological behavior after melting the formed **SC** crystals. By melting the SC crystals, the interface retains the presence of PDLA chains, which exert a compatibilization effect similar to that of PLLA chains. This difference in behavior before and after melting of the SC crystals at 250 °C clearly demonstrates the thermoreversible character of the used compatibilization approach.

At the elevated temperature of 250 °C, one might posit that the cocontinuous structure could be changed due to its unstable thermodynamic state. To assess the persistence of the cocontinuous structure, Han’s approach is employed.^65−67^ It has been proven that a deviation from a slope of 2 in the terminal region and the existence of temperature independency in the Han plots suggests the persistence of the cocontinuous structure before and after temperature variations.^67^ In addition, the effect of SC crystals on the viscoelastic properties becomes more evident when examining the correlation between the storage modulus and the loss modulus of the blends both before and after annealing at 250 °C where the SC crystals are molten. Figure 8d depicts Han plots of the blends conducted at 200 °C before and after annealing at 250 °C. The slopes of the curves deviate gradually from 2 at the terminal region (low frequencies) for all the samples due to the interface structure of the cocontinuous morphology in which *G*′is higher than *G*″ at low frequencies. At a given *G*″, the PVDF/SAD/PLLA blend exhibits the highest values of *G*′ before melting the SC crystals due to its extra elasticity contribution. Furthermore, the *G*′versus *G*″ plots after annealing at 250 °C suggest that both the compatibilized and uncompatibilized PVDF/PLLA blends exhibit the same type of microstructure (cocontinuous), as the curves are nearly identical for all blends.

### Effect of Stereocomplexation at the Interface on the Mechanical Properties

The unique cocontinuous structure allows the properties of the interpenetrating phases to be combined, thereby enhancing properties such as the mechanical performance of the final material when compared to the individual phases. Incorporating PVDF, known for its ductile nature, in a more brittle PLLA contributes to improved ductility of the blend. However, a crucial condition for this enhancement is the establishment of a reinforced interface between the phases, facilitating the transfer of stresses. Accordingly, compatibilizing the blend is crucial to improving the PVDF/PLLA blend interface. The effect of compatibilizers on the mechanical properties, with a specific focus on SC crystal formation at the interface, is investigated by using dynamic mechanical thermal analysis (DMTA) and tensile tests.

**Figure 9:**
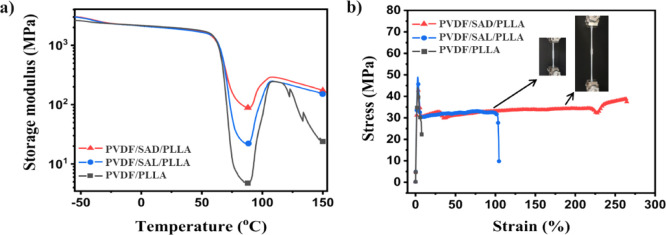
(a) DMTA thermograms and (b) selected tensile stress–strain curves of pure PVDF/PLLA blends and blends with various compatibilizers, repeat measurements of tensile tests are plotted in Figure S7. Inset shows the images of the PVDF/SAD/PLLA sample extending during the test.

In Figure 9a, the storage modulus measured by DMTA is plotted against the temperature, revealing multiple transitions across all samples. The decreases observed at −40 °C and around 70 °C correspond to the glass transition temperatures (*T*_g_) of PVDF and PLLA, respectively. In the pure blend, the continuous PLLA phase softens above its glass transition temperature (74.5 °C) and becomes mobile with further temperature increase, resulting in a decreased modulus around *T*_g_. However, beyond this temperature, cold crystallization occurs which results in a subsequent increase in storage modulus,^10,11,14^ The same behavior can be seen for the compatibilized samples. It should be noted that the samples were prepared under faster cooling conditions than the cooling rate of 10 °C/min used for the DSC analysis. This discrepancy explains the observed cold crystallization behavior during heating in the DMTA measurements. The effect of the compatibilizers on the *T*_g_ values of PLLA and PVDF in the pure and compatibilized blends is evaluated by determining the *T*_g_ values from the maximum in tan δ in DMTA results (Figure S6), with the *T*_g_’s summarized in Table S1. As can be seen, adding SAL and SAD does not significantly change *T*_g,PVDF_ and *T*_g,PLLA_ in the blends. From this observation, it can be assumed that there are no significant unwanted reactions that hinder chain mobility in each phase.

The PVDF/SAD/PLLA blend exhibits a significant enhancement in modulus around the PLLA glass transition temperature and at higher temperatures compared to PVDF/SAL/PLLA and pure PVDF/PLLA. The PVDF/SAL/PLLA also exhibits a higher modulus around the PLLA softening point compared to the PVDF/PLLA blend. The presence of SAL at the interface establishes a connection between the PVDF and PLLA phases, allowing PVDF to influence the mechanical properties. On the other hand, the presence of SC crystals at the interface in PVDF/SAD/PLLA results in a remarkable increase in the minimum storage modulus (88.7 MPa), which is around 4 times and 19 times higher than that of the blend with SAL and without compatibilizer, exhibiting minimum modulus values of 21.9 and 4.7 MPa, respectively.

The stress–strain curves of blends featuring SAL and SAD compatibilizers, along with the pure blend, reveal yielding behavior in all samples (Figures 9b and S7 ). The PVDF/PLLA blend without compatibilizers exhibits brittleness and weakness, with an elongation at a break of approximately 6% and a tensile strength of 30–40 MPa. In contrast, the SAL compatibilized sample shows an improved performance, with a strain at break between 40% and 101% and a strength of about 31 MPa. Particularly, the SAD-compatibilized blend, exhibiting ICIC, demonstrates a significant enhancement in elongation at break, reaching between 160% and 264%, and a strength at break of about 38 MPa. These elongation values for the PVDF/SAD/PLLA blend are 28 and 1.5 times higher than those of the neat PVDF/PLLA blend and PVDF/SAL/PLLA blend, respectively. The same trend of mechanical property improvement was reported by Wang et al.,^12^ where the PVDF/PLLA blend system was compatibilized using PDLA grafted onto MMA (methyl methacrylate). They reported that for a PVDF/PLLA 50/50 wt % blend, adding 3 wt % of the compatibilizer (MG-*g*-PDLA) resulted in a cocontinuous morphology. The elongation at break was about 275%, and E′ in the range of 60–110 °C improved with the addition of this copolymer from 39.8 MPa for the pure blend to 261.4 MPa. This is because blends with SC at the interface can be self-supporting even when the PLLA phase softens, as the cocontinuous morphology is maintained by the SC thin layer during melt processing. Accordingly, the remarkably enhanced compatibility between PVDF and PLLA, coupled with the strengthened interface due to stereocomplexation, facilitates efficient stress transfer between PLLA and PVDF phases, resulting in a substantial increase in the ductility and the minimum storage modulus near the glass transition temperature of the PLLA.

**Figure 10:**
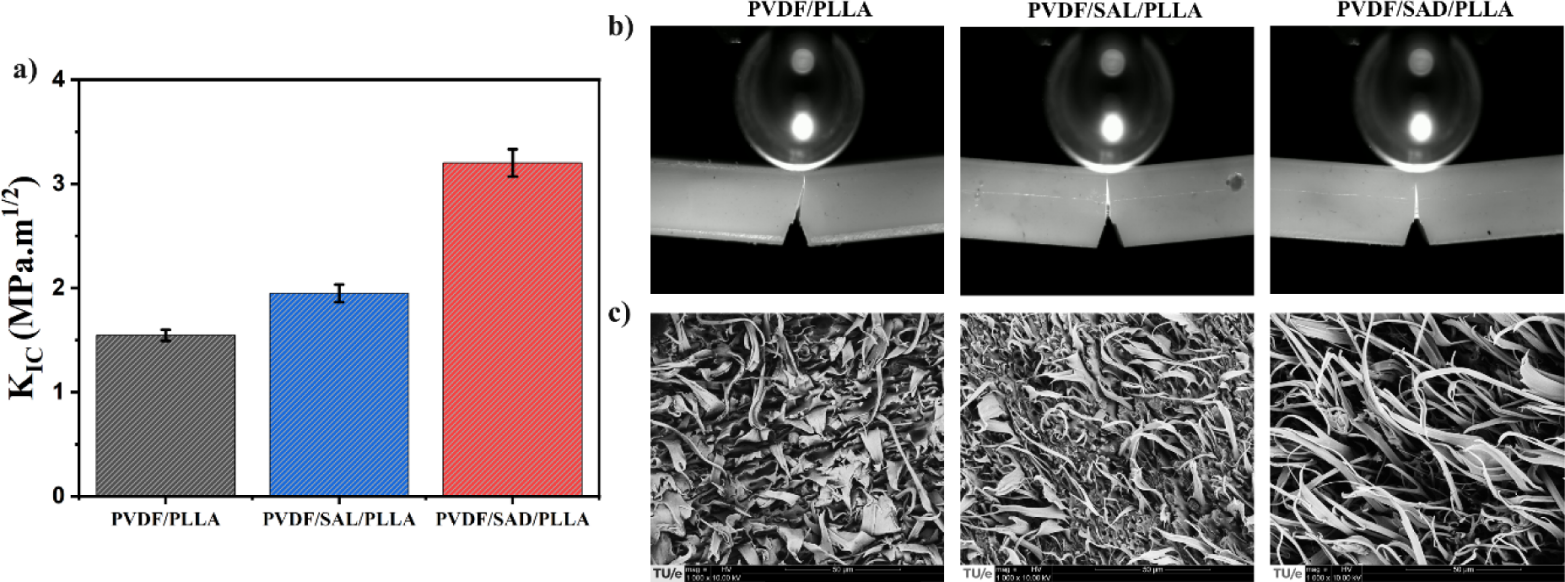
(a) The calculated fracture toughness factor (K_IC_), (b) images of the crack tip zone and the propagated crack at the surface of the SENB specimens, and (c) SEM images of the fractured surfaces of the blends. The error bars in (a) indicate the standard deviation.

The change in ductility of the blends is also evaluated by comparing their tensile toughness values (see Figure S7d). These values are determined from the area under the stress–strain curves obtained during tensile tests. The toughness value of the SAD-compatibilized blend is dramatically higher than those of pure PVDF/PLLA and the SAL-compatibilized blend. The fracture toughness of the pure and compatibilized blends is investigated in Figure 10a by comparing the fracture toughness factor (K_IC_) using single edge notch bending (SENB) tests (See Figure S8). The K_IC_ value indicates the material’s ability to hinder the initiation and propagation of cracks leading to fracture. The obtained K_IC_ values for the blends are as follows: PVDF/PLLA = 1.5 ± 0.05 MPa·m^1/2^, PVDF/SAL/PLLA = 1.9 ± 0.08 MPa·m^1/2^, and PVDF/SAD/PLLA = 3.2 ± 0.13 MPa·m^1/2^. These results reveal a drastic improvement in the fracture toughness and ductility of the PVDF/PLLA blend due to the SAD copolymer. The possible reason for the significant increase in the ductility of the SAD blend is the increase in the ability to undergo permanent plastic deformation up to fracture.

The SEM images of the fractured surfaces are shown in Figure 10c. The addition of SAL and SAD compatibilizers reduces the fibrillar sizes compared to those of the pure blend, indicating improved interfacial adhesion and stress transfer. The SAD-compatibilized blend exhibits even finer and more long-stretched fibrillar structures. This improved ductility and the pronounced reduction in fibril size are attributed to the rigid interfacial SC crystals, which not only strengthen the interface but also effectively restrict the coarsening of the phases, leading to a more refined and stable morphology.

## Conclusion

In this study, the concept of interface crystallization-induced compatibilization (ICIC) is investigated in a cocontinuous PLLA/PVDF system via stereocomplex (SC) crystal formation at the blend interface. The impact of the crystalline layer formation at the interface on morphology, thermal behavior, mechanical properties, and crystallization behavior is examined by comparing pure blends with systems employing a compatibilizer with the potential for stereocomplexation against those without this ability. Notably, the crystalline layer forms at the interface, particularly in the case of using poly(styrene-*co*-glycidyl methacrylate)-*graft*-poly(d-lactic acid) (SAD) where PDLA segments of the compatibilizer interact with PLLA chains of the PLLA phase to form SC crystals. The confirmed miscibility of each block of the SAD with the relevant phases in the blend ensures the formation of the SC crystal layer at the blend interface with a thickness of approximately 200–300 nm, as observed by AFM and nano-IR spectroscopy. This marks the first instance of detecting the SC layer using this technique and is associated with the most pronounced compatibilization effect evidenced by the reduction in domain size, changes in viscoelastic behavior, and the improvement in thermomechanical properties.

The formation of SC crystals at the interface enhances the stability of the cocontinuous structure and elevates the crystallization temperature of PLLA. This effect is attributed to the phenomenon of interfacial nucleation, which accelerates the overall crystallization process within the PLLA/PVDF blend. The cocontinuous structure, coupled with strong interfacial adhesion resulting from ICIC through stereocomplexation, facilitates efficient stress transfer among the phases in the blend. This phenomenon significantly enhances the tensile properties with a strain at break increase of up to 250%, doubles the fracture toughness factor (K_IC_) from 1.5 MPa·m^1/2^ to 3.2 MPa·m^1/2^ compared to the uncompatibilized blend, and also improves thermomechanical properties. The outcomes of this study suggest that the ICIC strategy holds promise for enhancing the thermal and rheological properties of PLA-based blends with a balanced tensile and fracture toughness that can be extended to other immiscible polymer blends. In conclusion, the elucidation of the crystalline layer location and distribution in the ICIC opens promising avenues for designing and achieving high-performance polymer blends.

## Supporting Information Available

(1) Additional materials and methods information for NMR spectroscopy, quasi-static fracture tests, and atomic force microscopy (AFM) measurements; (2) FTIR spectra of PDLA, SAN-*g*-GMA (SAG), and SAG-*g*-PDLA (SAD); (3) FTIR spectra of PLLA, SAN-*g*-GMA (SAG), and SAG-*g*-PLLA (SAL); (4) FTIR spectra of PDLA, PVDF, SAN-*g*-GMA (SAG), and SAG-*g*-PDLA (SAD) formed in the PVDF phase; (5) NMR spectra of PDLA, SAN-*g*-GMA (SAG), and SAG-*g*-PDLA (SAD); (6) H NMR and ^13^C NMR chemical shifts of different components; (7) AFM images of pure and compatibilized PVDF/PLLA blends; (8) glass transition temperatures of the components in the pure and compatibilized PVDF/PLLA blends; (9) evolution of loss factor (tan δ) of the components with temperature, obtained from DMTA tests; (10) tensile stress–strain curves of the blend specimens; (11) fracture behavior of the pure and compatibilized PVDF/PLLA blends; (12) the single edge notch bending (SENB) specimen dimensions; (13) references (PDF)
