# Supplementary material for: Toughening Immiscible Polymer Blends: The Role of Interface-Crystallization-Induced Compatibilization Explored Through Nanoscale Visualization
Source: ACS Appl Mater Interfaces. 2024 Oct 16;16(43):59174–87. doi: 10.1021/acsami.4c10829 (PMC11533176; doi:10.1021/acsami.4c10829)
Supplement: Supplementary file 1 — am4c10829_si_001.pdf [file am4c10829_si_001.pdf]

Supporting Information

Toughening Immiscible Polymer Blends: The Role of Interface-Crystallization-Induced Compatibilization Explored Through Nanoscale Visualization

Hamid Ahmadi [a], *Paul M. H. van Heugten* [a], *Alexander Veber* [b,c], *Ljiljana Puskar* [c], *Patrick D. Anderson* [a], *Ruth Cardinaels* [*a,d]

[a] Processing and Performance of Materials, Department of Mechanical Engineering, Eindhoven University of Technology, P.O. Box 513, 5600 MB Eindhoven, The Netherlands

[b] Department of Chemistry, Humboldt-Universität zu Berlin, Brook-Taylor-Straße 2, 12489 Berlin, Germany

[c] Institute for Electronic Structure Dynamics, Helmholtz-Zentrum Berlin für Materialien und Energie GmbH, Albert-Einstein-Straße 15, 12489 Berlin, Germany

[d] Soft Matter, Rheology and Technology, Department of Chemical Engineering, KU Leuven, Celestijnenlaan 200J, 3001 Leuven, Belgium

Email: R.M.Cardinaels@tue.nl

Section S1: Additional materials and methods information

NMR spectroscopy

Proton-decoupled H NMR spectra are recorded using a Bruker Avance III HD 400-MHz NMR spectrometer. ^{13}C NMR measurements are conducted using a Bruker Avance II+ 600-MHz NMR spectrometer. $CDCl_3$ purchased from sigma aldrich (99.8 atom%D, and 0.03% (v/v) is used as the solvent. Each H NMR and ^{13}C NMR experiment is performed on a solution containing around 10 mg and 35-38 mg of sample, respectively.

Quasi-static fracture tests

The fracture toughness (K_{IC}) is determined using the following equation according to ASTM E1820:

$$K_{IC} = \frac{F_{max} * L_s}{B * W^{3/2}} * f\left(\frac{a}{W}\right)$$

$$f\left(\frac{a}{W}\right) = \frac{3\left(\frac{a}{W}\right)^{1/2}\left[1.99 - \left(\frac{a}{W}\right) * \left(1 - \left(\frac{a}{W}\right)\right) * \left(2.15 - 3.93\left(\frac{a}{W}\right) + 2.7\left(\frac{a}{W}\right)^2\right)\right]}{2\left(1 + 2\left(\frac{a}{W}\right)\right) * \left(1 - \left(\frac{a}{W}\right)\right)^{3/2}}$$

$$L_s = L - 2l$$

$$a = a_0 + p$$

Here F_{max} is the maximum load, f(a/W) is the geometrical factor, W is the width, L_s is the span distance, B is the specimen thickness and a is the notch length (see Figure S9).

Atomic force microscopy (AFM)

For the AFM image shown in Figure S5b, a cryo-microtome section of the PVDF/SAD/PLLA blend is prepared. The imaging is conducted using an AFM (Digital Instrument Nanoscope IIIA Multimode) in tapping mode. A Silicon cantilever (model ACTA-SS from APP NANO) with an elastic constant of 25-75 N/m and a resonance frequency range of 200-400 Hz is employed. Before imaging, the sample is microtomed at -20°C using a Leica RM2165 microtome with a Leica LN21 cryo-unit and a diamond knife, then mounted on a custom-made AFM holder for the measurement.

Section S2: Additional data

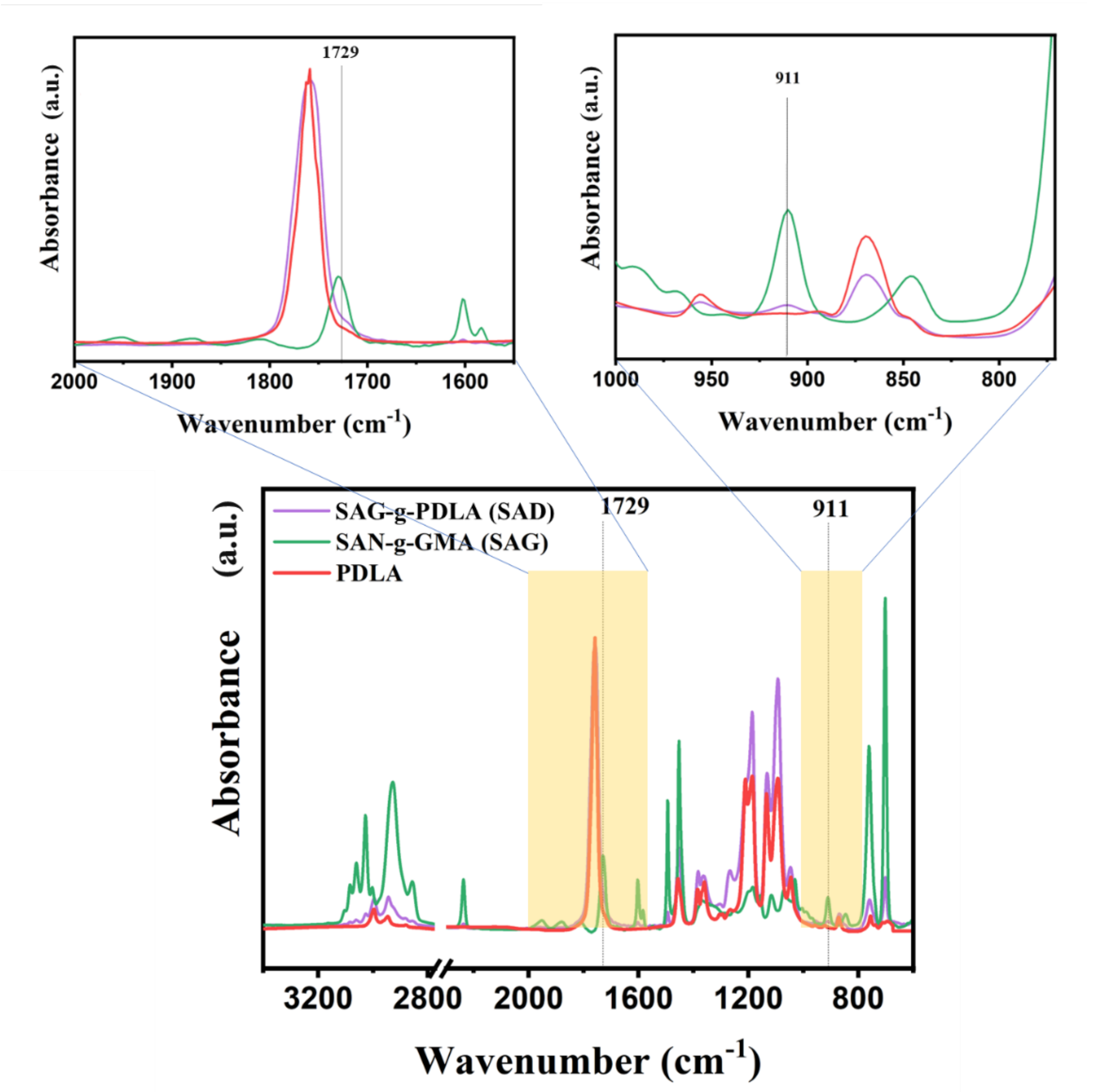

Figure S1: FTIR spectra of PDLA, SAN-g-GMA (SAG), and SAG-g-PDLA (SAD).

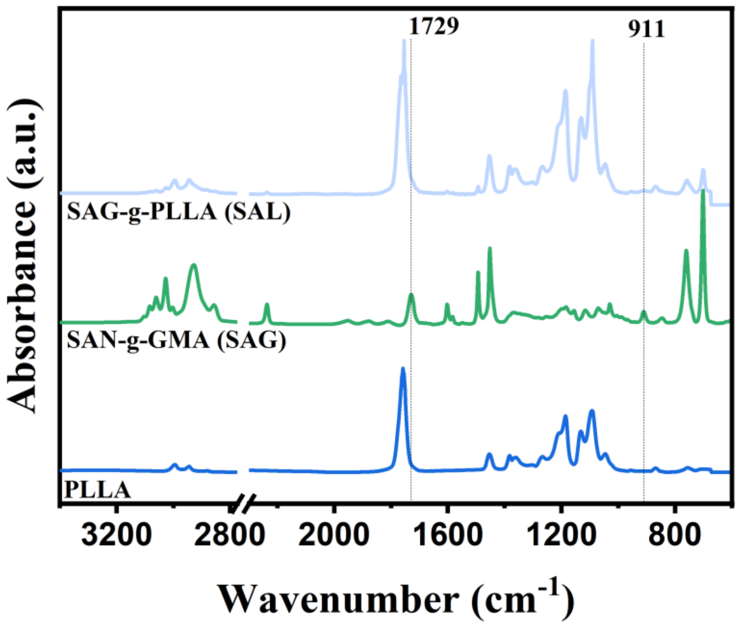

Figure S2: FTIR spectra of PLLA, SAN-g-GMA (SAG), and SAG-g-PLLA (SAL).

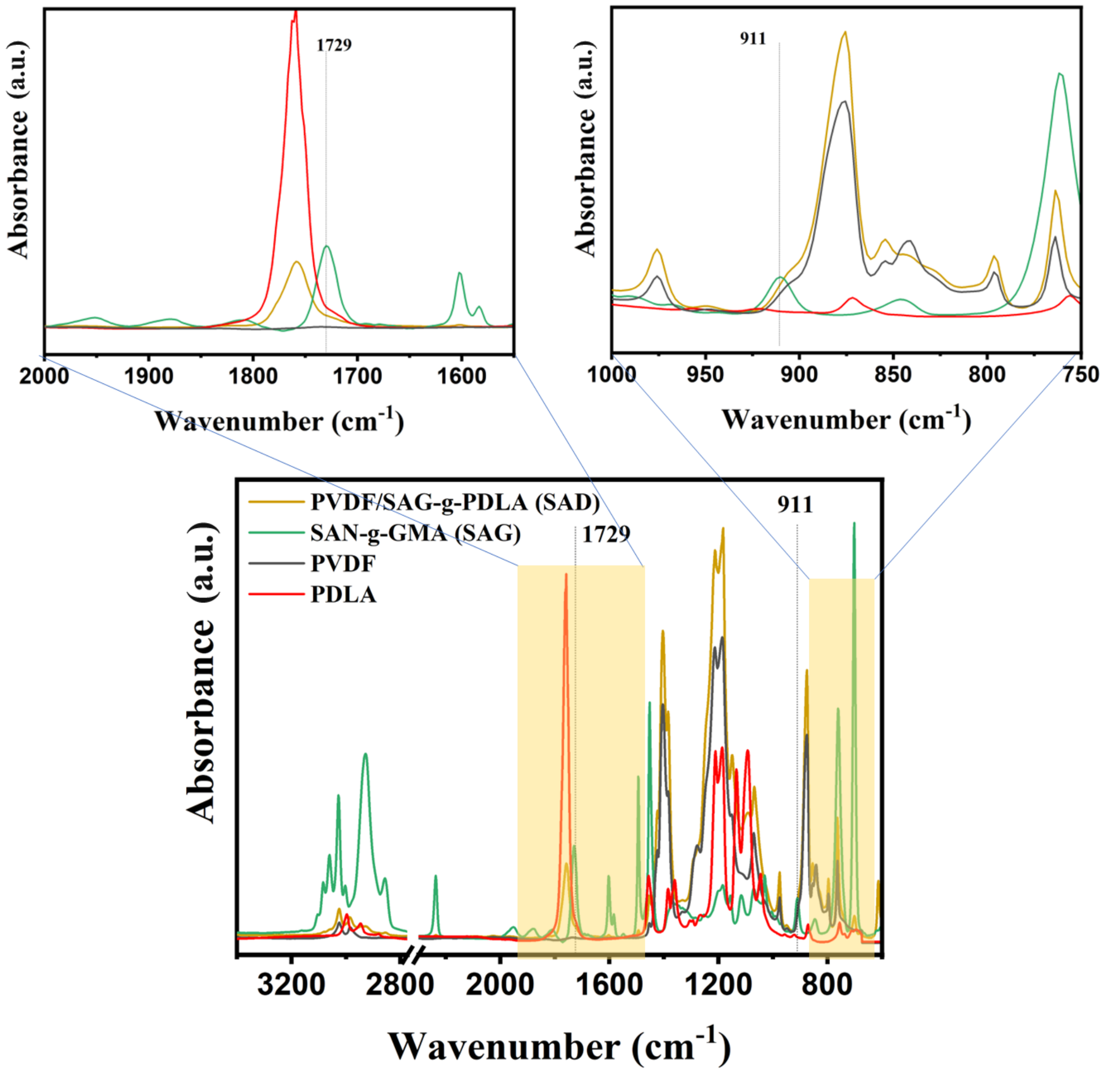

Figure S3: FTIR spectra of PDLA, PVDF, SAN-g-GMA (SAG), and SAG-g-PDLA (SAD) formed in the PVDF phase.

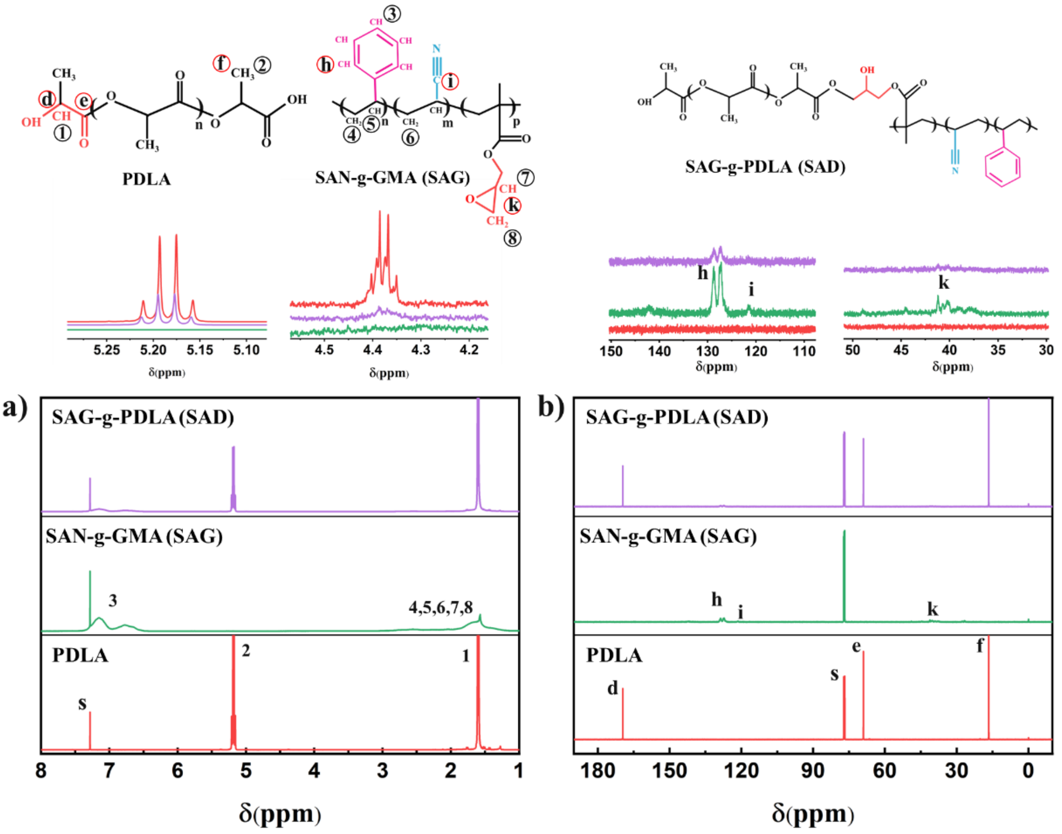

Figure S4: a) ^{1}H NMR , and b) ^{13}C NMR spectra of PDLA, SAN-g-GMA (SAG), and SAG-g-PDLA (SAD). The chemical structures, highlighting the assigned H and C atoms from the NMR measurements, along with a magnified section of the corresponding results, are shown at the top of the images. The letter "s" indicates the chemical shift of the solvent.

In the ^{1}H-NMR spectrum (Figure S4a), the signals at 1.6 ppm and 5.2 ppm represent the methyl and methine protons of PDLA, respectively (1 and 2 in Figure S4a) [1]. The aromatic protons of styrene appear between 6.5–7.5 ppm (3 in Figure S4b), while the signals between 1 ppm and 3 ppm represent the backbone protons of acrylonitrile and styrene (4, 5, and 6 in Figure S4b) [2]. The signals between 2.6 ppm and 2.8 ppm correspond to the methyl and methine protons of the epoxy group in GMA (7 and 8 in Figure S4a)[1].

The disappearance of the signal corresponding to the methine proton at the α-position relative to the terminal –OH group of PDLA, around 4.4 ppm, indicates the substitution of PDLA's terminal hydroxyl groups with GMA. This confirms the formation of the SAG-g-PDLA (SAD) copolymer[3].

For pure PDLA, the ^{13}C NMR spectrum displays characteristic chemical shifts at 169 ppm for the carbonyl carbon (CO) (d in Figure S4b), between 68.2 and 69.8 ppm for the methine carbon (CH) (f in Figure S4b), and between 14 and 18 ppm for the methyl carbon (CH_3) (e in Figure S4b)[4]. For the SAN-g-GMA component, the aromatic carbons (C_6H_5-) of styrene appear around

127-128 ppm (h in Figure S4b), the nitrile carbon (CN) displays a peak around 120 ppm (i in Figure S4b), and the epoxy ring carbons of GMA show a peak around 45 ppm (k in Figure S4b)[5].

To further estimate the extent of grafting, we calculated the grafting ratio by comparing the integral values of the characteristic peaks in the ^{13}C NMR spectrum. The grafting ratio was determined using normalized integrals according to the amount of carbon of each component, from the ^{13}C NMR spectra to calculate the mole fractions of styrene, acrylonitrile, GMA, and PLA in both pure SAN-g-GMA (SAG) and the SAG-g-PDLA (SAD). The mole fractions, along with the molecular weights of SAG and PDLA, and the mass of the sample (35–38 mg), were used to calculate the moles of each component in the SAN-g-GMA and SAG-g-PDLA. Given that the SAD sample was not purified, the mixture contained both reacted and unreacted components. The reaction was assumed to occur primarily at the GMA segment of SAN-g-GMA, with the blend composition being 95% PDLA and 5% SAN-g-GMA. The moles of unreacted GMA were estimated based on the composition of pure SAN-g-GMA in the 95/5 PDLA/SAN-g-GMA blend and directly measured from the NMR spectrum of the SAG-g-PDLA sample. The grafting ratio was calculated as:

Grafting ratio= (Moles of GMA from 5% pure SAN-g-GMA-Moles of unreacted GMA in the SAD)/(Total moles of PLA in the mixture)

Based on this approach, the grafting ratio was determined to be 4.7%.

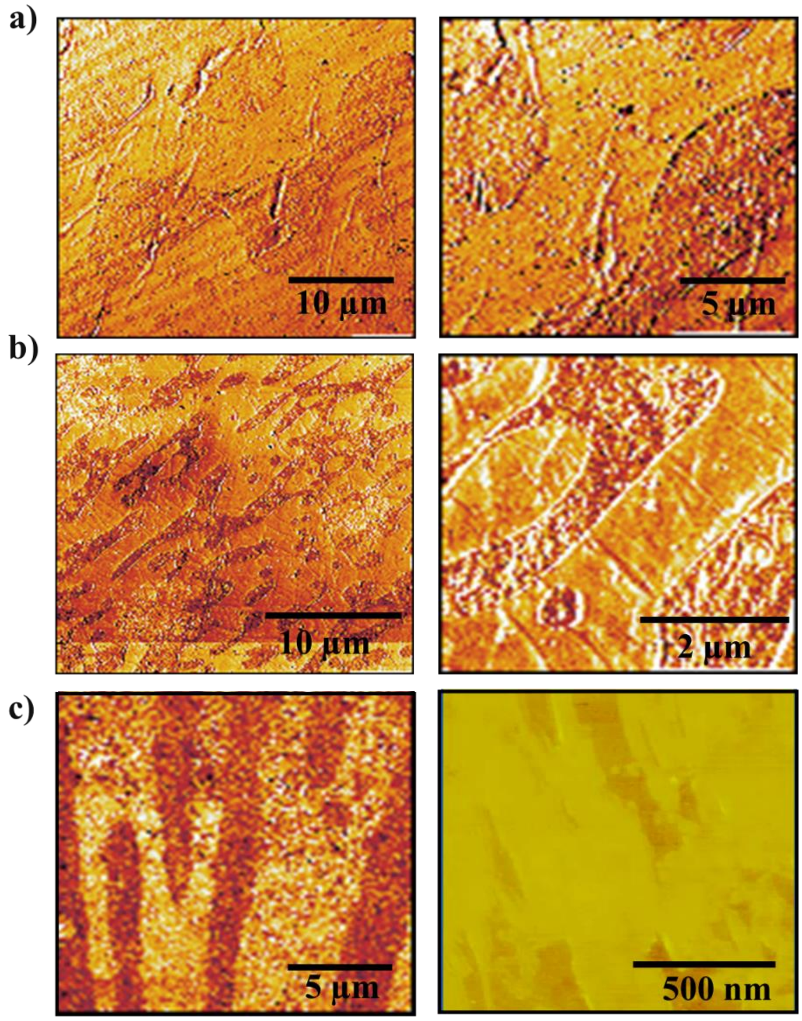

Figure S5: AFM images of a) pure PVDF/PLLA blend, b) with SAL compatibilizer (PVDF/SAL/PLLA), c) with SAD compatibilizer (PVDF/SAD/PLLA blends). The light and dark colors correspond to the PLLA and PVDF phases, respectively**.**

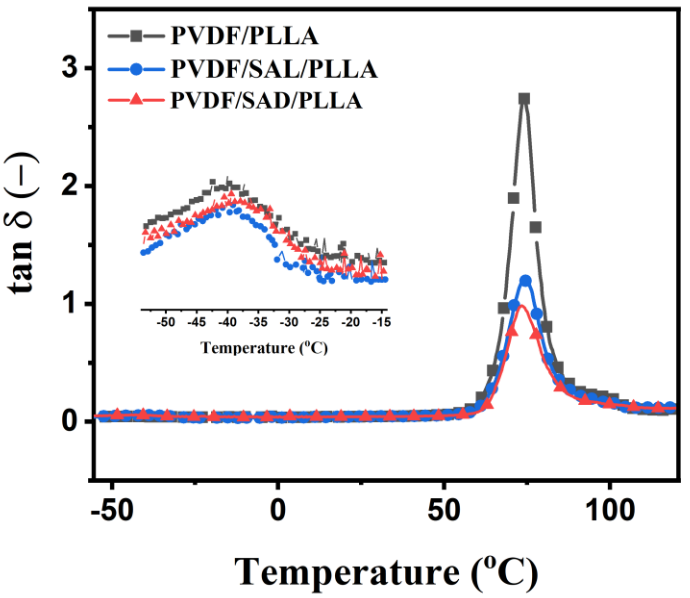

Figure S6: Evolution of loss factor tan δ with temperature, obtained from DMTA tests, for PVDF/PLLA blends and blends with various compatibilizers.

Table S1: Glass transition temperatures of PVDF ($T_{g,PVDF}$) and PLLA ($T_{g,PLLA}$) in the pure and compatibilized PVDF/PLLA blends.

Sample name	$T_{g,PLLA}$ (°C)	$T_{g,PVDF}$ (°C)
PVDF /PLLA	74.5	39.6
PVDF/SAL/PLLA	74.8	39.1
PVD/SAD/PLLA	73.6	38.1

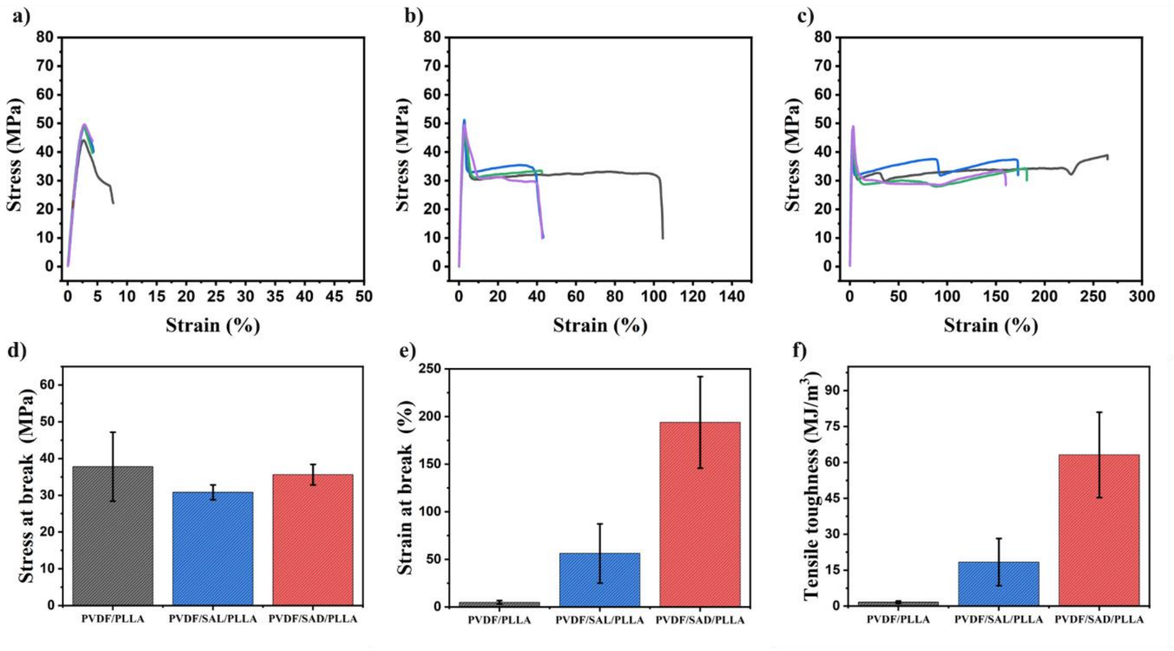

Figure S7: Tensile stress-strain curves of the blend specimens. a) pure PVDF/PLLA, b) PVDF/SAL/PLLA, c) PVDF/SAD/PLLA, d) stress at break, e) strain at break and f) tensile toughness of the pure and compatibilized blends. The error bars indicate the standard deviation.

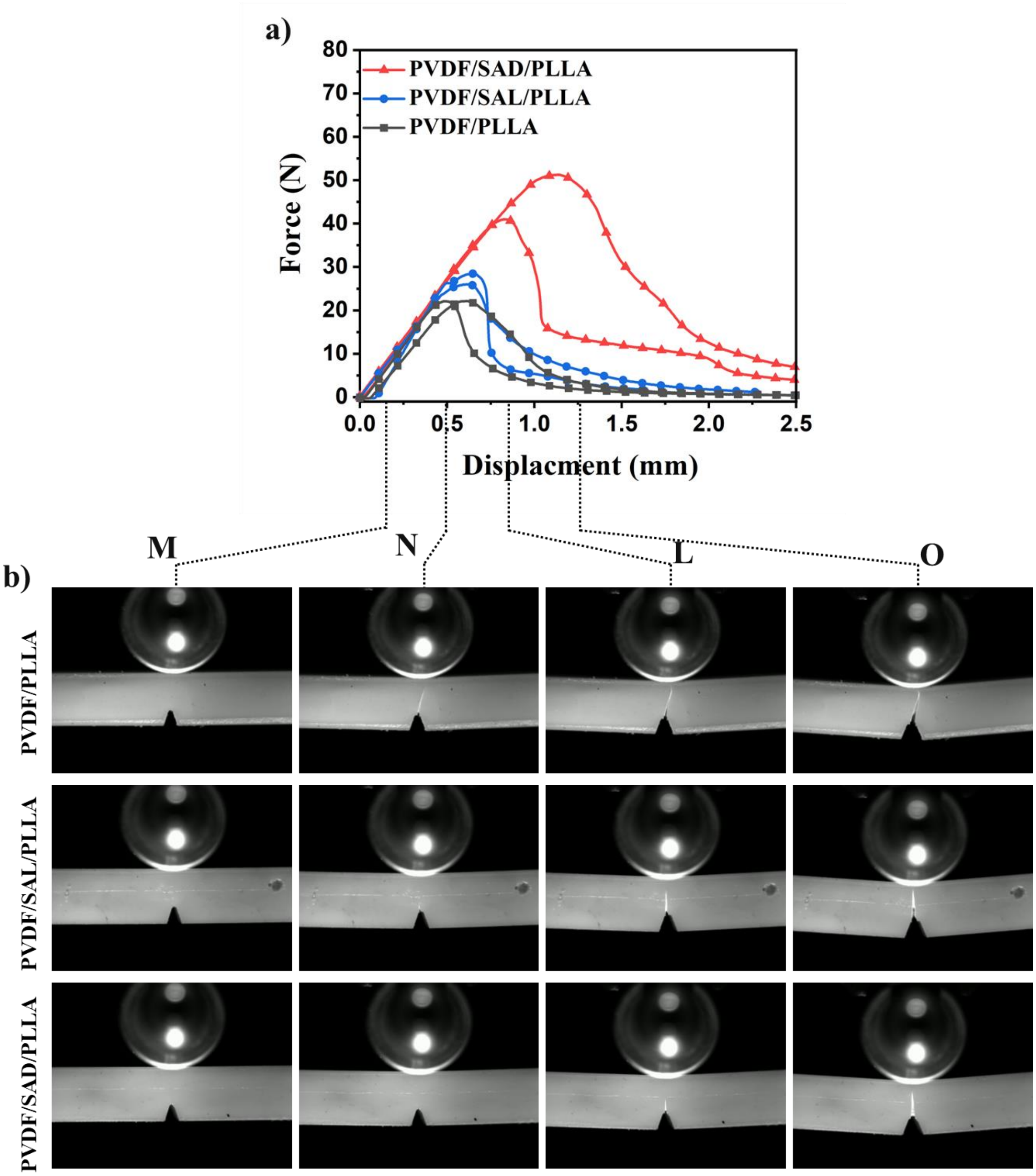

Figure S8: a) The measured indentation load versus indenter displacement in the quasi-static fracture tests, b) Images of the crack tip zone and the growing crack at the surface of the SENB specimen during different stages (M=0.1 mm, N=0.5 mm, L=0.8 mm, and O=1.2 mm) of the SENB test for the PVDF/PLLA blend and blends with various compatibilizers.

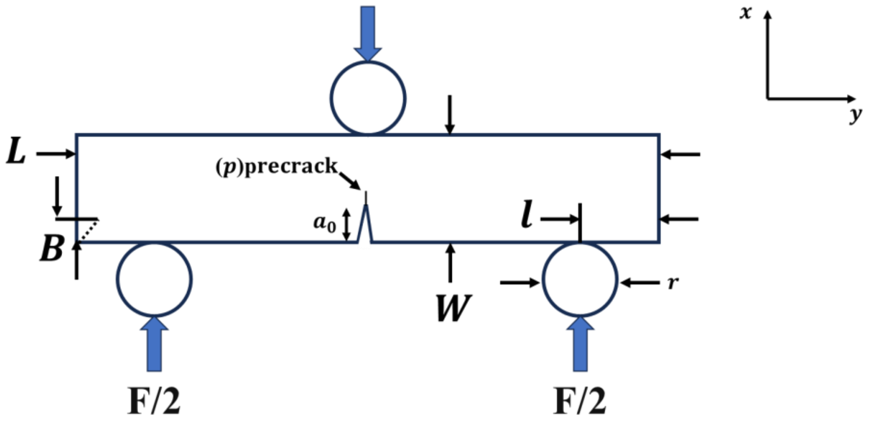

Figure S9: The single edge notch bending (SENB) specimen dimensions: Thickness B = 4.8 mm, L = 55.5 mm, l = 6.5 mm, W = 5.3 mm, $a_0 \approx 1.5$ mm , $p \approx 0.3$ mm. The radius r of the cylindrical supporting rollers and indenter is equal to 10 mm.

References

1. Deng, S. H.; Bai, H. W.; Liu, Z. W.; Zhang, Q.; Fu, Q., Toward Supertough and Heat-Resistant Stereocomplex-Type Polylactide/Elastomer Blends with Impressive Melt Stability via Formation of Graft Copolymer during One-Pot Reactive Melt Blending. *Macromolecules* **2019,** *52* (4), 1718-1730.

2. Alam, M. M.; Peng, H.; Jack, K. S.; Hill, D. J.; Whittaker, A. K. J. J. o. P. S. P. A. P. C., Reactivity Ratios and Sequence Distribution Characterization by Quantitative 13C NMR for RAFT Synthesis of Styrene-Acrylonitrile Copolymers. **2017,** *55* (5), 919-927.

3. Bagheri, M.; Motirasoul, F. J. J. o. P. R., Synthesis, characterization, and micellization of cholesteryl-modified amphiphilic poly (L-lactide)-block-poly (glycidyl methacrylate) as a nanocarrier for hydrophobic drugs. **2013,** *20*, 1-9.

4. Suganuma, K.; Horiuchi, K.; Matsuda, H.; Cheng, H.; Aoki, A.; Asakura, T. J. M., Stereoregularity of poly (lactic acid) and their model compounds as studied by NMR and quantum chemical calculations. **2011,** *44* (23), 9247-9253.

5. Kim, I. C.; Kwon, K. H.; Kim, W. N. J. J. o. A. P. S., Gloss reduction and morphological properties of polycarbonate and poly (methyl methacrylate-acrylonitrile-butadiene-styrene) blends with SAN-co-GMA as a reactive compatibilizer. **2018,** *135* (27), 46450.
